# Altered cerebrovascular response to acute exercise in patients with Huntington’s disease

**DOI:** 10.1093/braincomms/fcaa044

**Published:** 2020-04-16

**Authors:** Jessica J Steventon, Hannah Furby, James Ralph, Peter O’Callaghan, Anne E Rosser, Richard G Wise, Monica Busse, Kevin Murphy

**Affiliations:** f1 Cardiff University Brain Research Imaging Centre, School of Physics and Astronomy, Cardiff University, Cardiff CF24 4HQ, UK; f2 Neuroscience and Mental Health Research Institute, School of Medicine, Cardiff CF24 4HQ, UK; f3 Cardiff University Brain Research Imaging Centre, School of Psychology, Cardiff University, Cardiff CF24 4HQ, UK; f4 Cardiology Department, University Hospital of Wales, Cardiff CF14 4XW, UK; f5 Cardiff Brain Repair Group, School of Biosciences, Cardiff University, Cardiff CF10 3AX, UK; f6 Centre for Trials Research, Cardiff University, Cardiff CF14 4YS, UK

**Keywords:** neurodegeneration, plasticity, cerebral blood flow, MRI

## Abstract

The objective of this study was to determine whether a single session of exercise was sufficient to induce cerebral adaptations in individuals with Huntington’s disease and to explore the time dynamics of any acute cerebrovascular response. In this case–control study, we employed arterial-spin labelling MRI in 19 Huntington’s disease gene-positive participants (32–65 years, 13 males) and 19 controls (29–63 years, 10 males) matched for age, gender, body mass index and self-reported activity levels, to measure global and regional perfusion in response to 20 min of moderate-intensity cycling. Cerebral perfusion was measured at baseline and 15, 40 and 60 min after exercise cessation. Relative to baseline, we found that cerebral perfusion increased in patients with Huntington’s disease yet was unchanged in control participants in the precentral gyrus (*P* = 0.016), middle frontal gyrus (*P* = 0.046) and hippocampus (*P* = 0.048) 40 min after exercise cessation (+15 to +32.5% change in Huntington’s disease participants, −7.7 to 0.8% change in controls). The length of the disease‐causing trinucleotide repeat expansion in the huntingtin gene predicted the change in the precentral gyrus (*P* = 0.03) and the intensity of the exercise intervention predicted hippocampal perfusion change in Huntington’s disease participants (*P* < 0.001). In both groups, exercise increased hippocampal blood flow 60 min after exercise cessation (*P* = 0.039). These findings demonstrate the utility of acute exercise as a clinically sensitive experimental paradigm to modulate the cerebrovasculature. Twenty minutes of aerobic exercise induced transient cerebrovascular adaptations in the hippocampus and cortex selectively in Huntington’s disease participants and likely represents latent neuropathology not evident at rest.

## Teaser

Nineteen participants with Huntington’s disease and 19 controls underwent MRI before and immediately after a 20-min exercise intervention. A region of interest analysis found that cerebral blood flow was selectively increased in participants with Huntington’s disease in the precentral gyrus, middle frontal gyrus and hippocampus 40 min after exercise cessation, suggesting that exercise exposes latent Huntington’s disease pathology.

## Graphical Abstract

**Graphical Abstract:**
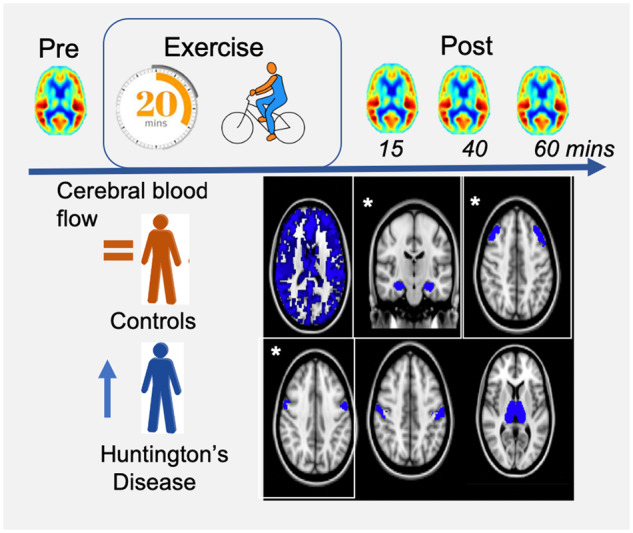

## Introduction

Huntington’s disease is a progressive neurodegenerative disorder caused by the expansion of a polyglutamine stretch within the Huntingtin gene (Gusella *et al.*, 1993; Vonsattel and DiFiglia, 1998). Neuropathology in Huntington’s disease causes cognitive dysfunction, psychiatric symptoms and a movement disorder characterized by involuntary movements and impaired motor control (Bates *et al.*, 2002). The availability of genetic testing means that at-risk Huntington’s disease family members can be identified early in the disease course and prior to the onset of symptoms. As a result, there is a drive to discover the earliest signs of neuropathology that can guide future therapeutics.

Current evidence suggests that long-term exercise interventions can produce functional improvements in a range of populations. Exercise studies using genetic mouse models of Huntington’s disease have produced promising results, with converging evidence showing an improvement, or delay, in the emergence of motor impairments (van Dellen *et al.*, 2000, 2008; Pang *et al.*, 2006; Harrison *et al.*, 2013; Herbst and Holloway, 2015). Similarly, a systematic review of 18 Huntington’s disease patient studies found evidence for beneficial effects of exercise on motor function, gait speed and balance (Fritz *et al.*, 2017). Despite this, the mechanisms supporting these exercise-induced functional improvements in Huntington’s disease are unclear.

Evidence for a beneficial effect of exercise on Huntington’s disease neuropathology is largely limited to genetic rodent models of Huntington’s disease and remains equivocal. A reduction in striatal neuropathology has been observed in some studies (Pang *et al.*, 2006; Harrison *et al.*, 2013), whilst others have shown no effect of exercise on the degree of atrophy or mutant huntingtin protein (mHtt) aggregation (van Dellen *et al.*, 2008). In a small patient study, a 9-month multidisciplinary rehabilitation intervention that included exercise as a component increased brain volume in the grey matter, caudate and prefrontal cortex and improve cognition, although the independent contribution of exercise was not determined (Cruickshank *et al.*, 2015).

Inconsistencies across studies in the type, dose and duration of exercise likely contribute to discrepancies in the literature and may be masking the therapeutic potential of exercise in Huntington’s disease. To address this, a focused investigation of the temporal dynamics of exercise is needed. Emerging evidence suggests regional exercise effects on the brain may overlap in terms of acute and long-term timescales (Weng *et al.*, 2017). For example, in healthy adults, a single exercise session has been shown to improve motor function (Mang *et al.*, 2014; Skriver *et al.*, 2014), improve cognition (Winter *et al.*, 2007; Coles and Tomporowski, 2008; Chang *et al.*, 2012; Roig *et al.*, 2012; Jo *et al.*, 2019), increase peripheral neurotrophin and catecholamine biomarkers (Winter *et al.*, 2007; Mang *et al.*, 2014; Skriver *et al.*, 2014) and alter cerebral perfusion (Smith *et al.*, 2010; MacIntosh *et al.*, 2014; Steventon *et al.*, 2020). However, it is not clear whether the cerebrovascular response to the physiological provocation of exercise will be the same in a disease group where resting perturbations in the vascular system are present. In Huntington’s disease, structural and functional cerebral vascular abnormalities have been shown (Vis *et al.*, 1998; Lin *et al.*, 2013; Rahman *et al.*, 2013; Hua *et al.*, 2014; Drouin-Ouellet *et al.*, 2015; Hsiao *et al.*, 2015; St-Amour *et al.*, 2015) and aggregation of mutant Huntingtin is present in the neurovascular unit (Drouin-Ouellet *et al.*, 2015), which may affect the ability of the vascular system to respond to the demands of acute exercise.

The current study is the first to examine the cerebrovascular response to a single session of exercise in Huntington’s disease. Previous work has shown an altered peripheral response during and following exercise (Steventon *et al.*, 2018), with metabolic and cardiorespiratory deficits found to reduce exercise performance and affect exercise recovery.

Arterial-spin labelling (ASL) MRI is a quantitative, non-invasive method to measure tissue perfusion and uses magnetically labelled arterial blood water protons as an endogenous tracer. ASL MRI has been shown to be a reliable and repeatable method for quantifying tissue perfusion in aged and clinical populations (Kilroy *et al.*, 2014) and has been shown to be sufficiently sensitive to detect transient perfusion changes after exercise cessation in healthy populations (Smith *et al.*, 2010; MacIntosh *et al.*, 2014; Steventon *et al.*, 2020). In patients with stroke—a patient cohort where baseline cerebrovascular perturbations are evident—20 min of low- and moderate-intensity cycling resulted in an intensity-dependent change in perfusion in the somatosensory cortex using ASL MRI, along with an intensity-independent response in the basal ganglia (Robertson *et al.*, 2015).

Here, we examined the effect of exercise on the cerebrovasculature to establish if the acute response to a single session of exercise is different in Huntington’s disease. Our primary hypothesis was a differential effect of exercise on cerebral blood flow (CBF) in patients with Huntington’s disease compared to controls, based on the evidence of cerebrovascular abnormalities in Huntington’s disease (Drouin-Ouellet *et al.*, 2015) and an altered cardiorespiratory response to submaximal exercise (Steventon *et al.*, 2018).

## Materials and methods

**Figure 1:**
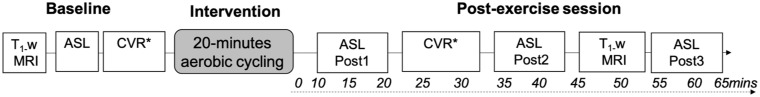
**Study design.** MRI measures were recorded up to 65-min after exercise cessation. Cycling was performed on an upright ergometer. *A scan to measure CVR using a breath-hold design was acquired but not analysed due to poor performance. CVR = cerebrovascular reactivity; T_1_-w = T_1_-weighted structural MRI scan acquired for image registration purposes.

The study design is detailed in Fig. 1.

### Participants

Huntington’s disease gene-positive participants [cytosine–adenine–guanine (CAG) length greater than or equal to 36, *n* = 19] were recruited from the South Wales Huntington’s disease research and management clinic, based in Cardiff, along with 19 healthy age-matched controls. Gene carriers included participants in the pre-manifest (*N* = 8) and manifest stages of disease (Stage I *N* = 3; Stage II *N* = 5; Stage III *N* = 3). Pre-manifest participants are those who have not yet met traditional motor criteria for a diagnosis of Huntington’s disease, based on a Diagnostic Confidence Level rating of ‘4’ according to the motor assessment section of the Unified Huntington’s disease Rating Scale. Manifest disease stages are based on the total functional capacity score on the Unified Huntington’s disease Rating Scale as defined previously (Shoulson and Fahn, 1979). Demographic and clinical data are shown in Table 1. All participants had a stable medication regime, defined as unchanged for 4 weeks. Exclusion criteria included any physical or psychiatric condition that would prohibit the participant from completing the exercise test, the inability to independently use the cycle ergometer, the inability to follow the protocol instructions, uncontrolled arterial hypertension, any neurological condition other than Huntington’s disease, pregnancy or childbirth in the last 6 weeks, current and/or history of cardiac, vascular or respiratory/pulmonary conditions, illicit drug use in the last 4 weeks and any MRI contraindications. Data were collected with ethical approval from Wales Research Ethics Committee (15/WA/0074), and all participants gave informed consent according to the Declaration of Helsinki. All Huntington’s disease gene carriers were registered on a global longitudinal study of patients with Huntington’s disease (Enroll-Huntington’s disease; REC no. 04/WSE05/89) and had been examined on the Unified Huntington’s disease Rating Scale in the 6-month period prior to scanning; thus, the clinical and genetic data were available.

**Table 1:** Sociodemographic, fitness, genetic and clinical data for Huntington’s disease and control participants

	Huntington’s disease (*n* = 19)	Healthy controls (*n* = 19)	*P-*value
Male gender, *N*	13	10	0.32
Age (years)	45.9 ± 2.2 (32–65)	42.2 ± 2.1 (29–63)	0.23
BMI (kg/m^2^)	26.2 ± 1.3 (18–40)	27.6 ± 0.9 (22–40)	0.41
IPAQ	2723.2 ± 847.1 (0–10755)^a^	2807.4 ± 682.8 (396–13488)	0.94
VO_2_ peak (ml/kg/min)	34.6 ± 2.5 (18–50)	37.4 ± 2.3 (25–61)	0.42
MAP (mmHg)^b^	92.70 ± 2.07	88.06 ± 2.77	0.19
CAG repeat length	43.7 ± 0.6 (41–50)	NA	
Disease burden [(CAG length_*n*_-35.5) × age]	370.3 ± 24.4 (208–575)		
UHDRS total motor score	25.7 ± 4.4 (0–68)		
UHDRS composite score (Schobel *et al*., 2017)	12.13 ±1.19 (2.8–18.6)		
UHDRS total functioning capacity	10.2 ± 0.8 (3–13)		

Mean ± SEM (range). One-way ANOVA or chi-squared tests were used to compare patients with Huntington’s disease to controls.

a One Huntington’s disease participant had an extreme high IPAQ score (353 512) that was removed from the group mean data to avoid skew.

b Rest measures calculated after 15-min supine rest.

BMI = body mass index; IPAQ = International Physical Activity Questionnaire; MAP = mean arterial pressure; NA = not applicable; UHDRS = Unified Huntington’s disease Rating Scale.

### Exercise intervention

The exercise intervention followed the procedures as described previously (Steventon *et al.*, 2020). Participants underwent 20 min of moderate-intensity aerobic cycling on an upright ergometer (Lode Ergometer, Lode, Groningen, Netherlands) at a prescribed intensity of 50–70% of the maximal heart rate reserve (HRR) determined using the Karvonen formula (American College of Sports Medicine, 2012).

During upright rest, the warm up and warm down period and at 3-min intervals during exercise, lactate concentration was collected, blood pressure was measured and self-report ratings of perceived exertion were recorded using a 10-point Borg scale (Borg, 1974). After 20 min, participants completed a 2-min warm down and then immediately returned to the MR scanner.

### MRI acquisition and image processing

MRI of the brain was performed on a 3 T whole-body MRI system (GE Excite HDx, Milwaukee, WI, USA) equipped with a body transmit and eight-channel receive head coil.

For the quantitative measurement of cerebral perfusion (ml/100 g/min) at baseline and at three time points post-exercise (Fig. 1), a PICORE Pulsed ASL sequence was performed (Wong *et al.*, 1997) with a gradient-echo spiral readout at eight inversion times [inversion time_1–8_ = 400, 500, 600, 700, 1100, 1400, 1700 and 2000 ms, two separate scan series, echo time = 2.7 ms, repetition time (TR) = variable, 15 slices (7 mm thick + 1.5 mm gap), slice delay = 52 ms, field of view (FOV) = 198 mm, 64 × 64 matrix = ∼3.1 mm^2^ in-plane resolution, 20 cm tag width]. A Quantitative Imaging of Perfusion with a Single Subtraction (Wong *et al.*, 1998) cut-off of the label was applied at 700 ms for inversion times >700 ms. To estimate the equilibrium magnetization (*M*_0_) of arterial blood, a single echo spiral k-space scan was acquired with the same parameters as above, minus the ASL tag preparation. A minimum contrast scan (echo time/TR = 11/2000 ms) was also acquired to correct for field inhomogeneity (Wu *et al.*, 2011).

A three-dimensional T_1_-weighted fast spoiled gradient-echo sequence was acquired at baseline and in the post-exercise scan session for image registration purposes (256 × 256, slice thickness = 1 mm giving a resolution of 1 mm^3^, TR/echo time 7.90/3.0 ms).

At baseline and 20 min post-exercise, a breath-hold challenge was performed to measure cerebrovascular reactivity (10 end-expiration breath-holds of 15-s length interleaved with 30 s periods of paced breathing) with a single-shot PICORE Quantitative Imaging of Perfusion with a Single Subtraction pulse sequence (Wong *et al.*, 1998). However, due to difficulties following the task instructions and poor performance of the breath-holds during acquisition, these data were not analysed (see Supplementary material).

CBF quantification followed the procedures described by Steventon *et al.* (2020). Data were analysed in AFNI (Cox, 1996) and FSL (Jenkinson *et al.*, 2012). Images were motion corrected, brain extracted, and an M_0,__CSF_ image was created following the procedures described by Warnert *et al.* (2015). The M_0_ for arterial blood was then calculated according to methods described by Wong *et al.* (1998) with CSF as a reference. Perfusion quantification was performed on a voxel-by-voxel basis using a two-compartment model (Chappell *et al.*, 2010) and employing partial volume correction (Chappell *et al.*, 2011) to address signal contamination associated with atrophy.

Median grey matter values were calculated for the four perfusion images (baseline, post-1, post-2, post-3) to assess global perfusion. Based on the exercise literature (Pereira *et al.*, 2007; Smith *et al.*, 2010; MacIntosh *et al.*, 2014), regional changes in perfusion were assessed in a number of *a priori* defined regions of interest (ROIs): the thalamus (a key hub for the motor system), the hippocampus (evidence for exercise-induced neurogenesis and blood flow changes; van Praag *et al.*, 2005; Pereira *et al.*, 2007; Steventon *et al.*, 2020) and three key cortical ROIs involved in sensorimotor processing—the middle frontal gyrus (MFG), postcentral gyrus and precentral gyrus. Perfusion was also examined in the caudate, based on the hallmark striatal pathology in Huntington’s disease (Paulsen *et al.*, 2010).

ROIs were segmented from the T_1_-weighted image acquired in the same scan session, and the median CBF values and arterial arrival time—the time taken for blood to travel from the labelling slab to the tissue (Wang *et al.*, 2003; Zappe *et al.*, 2007)—were calculated for each ROI.

### Cardiorespiratory measures

Pulse waveforms and oxygen saturation were recorded (MEDRAD, PA, USA), and blood pressure measurements were collected using an arm-cuff before and after each scan (OMRON, Tokyo, Japan). Expired gas content was recorded (AEI Technologies, PA, USA) and sampled at 500 Hz (CED, Cambridge, UK) to obtain measures of end-tidal partial pressure of carbon dioxide (PETCO_2_). A respiratory belt was placed just below the ribs to monitor ventilation. Mean arterial pressure was calculated as (systolic + 2 × diastolic)/3. Supine resting heart rate (HR) and blood pressure were measured after 15-min rest.

### Neuropsychological testing

To capture any functional cognitive gains, participants were invited to complete ‘baseline’ cognitive tests on a separate day within 1 month of completing the MRI session, before or after to control for order effects. The post-exercise tests were completed immediately after the second MRI scan, at 60–90 min after exercise cessation.

Five pencil and paper tasks were used to measure cognitive functioning: (i) The Forward Digit Span (Wechsler Adult Intelligence Scale Fourth Edition (WAIS-IV)) was used as a measure of verbal short-term memory. Participants were asked to repeat a digit sequence orally, beginning with two digits, and increasing in length by one digit following successful repetition of two lists of digits at a given length. (ii) The letter verbal fluency test was used to assess verbal functioning. Participants were required to produce words beginning with a certain letter of the alphabet in three respective 60-s trials. (iii) The Trail Making Test (part B) was used to measure visual attention, speed of processing, mental flexibility and executive functions. Participants were presented with stimuli consisting of randomly placed numbers and letters and were required to connect the numbers of letters in sequence and alternating order (e.g. 1-A-2-B). (iv) The Symbol Digit Modalities Test was used as a motor and psychomotor speed, with participants requiring to identify as many numbers (1–9) corresponding to a set of symbols in a single 90 s. (v) The Speed of Comprehension subtest from the speed and capacity of language processing test was used as a measure of processing speed; participants were required to determine if a list of sentences was correct or incorrect within a 2-min period. As motor speed may confound performance on many of the cognitive tests, participants completed a computer-based Speeded Tapping Test (Reitan, 1979) with their dominant hand, tapping the space bar as quickly as possible with their index finger. After 20 taps, the average number of taps per minute was calculated and averaged over three trials.

### Clinical and genetic predictor measures

The motor component (total motor score) of the Unified Huntington’s disease Rating Scale provides a summed score of motor function including chorea, dystonia, motor impersistence, gait, rigidity, bradykinesia, finger tapping and ocular movements. CAG repeat length was also used as a predictor.

### Baseline fitness test

To measure baseline fitness, participants underwent an incremental cycle ergometer exercise test (1000 W Cranlea; Human Performance Ltd, Birmingham, UK) on a separate day as described previously (Steventon *et al.*, 2018). The test protocol consisted of 2 min of rest, 2 min of unloaded cycling, followed by 25-W increments every 2 min, starting at 50 W. The exercise test was symptom limited; individuals were instructed to pedal until discomfort or fatigue prevented them from maintaining the required cadence. Pulmonary gas exchange was measured on a breath-by-breath basis (MetaMax 3B; Cortex Biophysik GmbH, Leipzig, Germany), with the gas analyser calibrated before each session according to the manufacturer’s instructions. HR was recorded continuously throughout using short-range telemetry (Polar S810, Finland). Breath-by-breath data were averaged every 30 s, and VO_2_ peak (peak oxygen uptake), the objective measure of cardiorespiratory fitness, was recorded as the average oxygen uptake across the final 15 s before the termination of the test. In addition, self-reported physical activity levels were recorded using the International Physical Activity Questionnaire (Craig *et al.*, 2003).

### Statistical analyses

An initial quality assessment examined any statistical outliers, defined as ˃3 SD from the group mean and removed them if found to be spurious (e.g. biologically implausible). To avoid biasing the results, all participants were included in the statistical analysis, including when missing data were present. Linear mixed effects models were used to assess change in cardiorespiratory physiology, cognition and cerebral perfusion for each *a priori* defined ROIs in R (version 1.1.463) (R Core Team, 2016) using the lme4 package (Bates *et al.*, 2015); between-subject effect: gene status (controls, Huntington’s disease), within-subject effect: time (baseline, post-1, post-2, post-3) and hemisphere for perfusion analysis (left and right); *P*-values were calculated from degrees of freedom estimated using Satterthwaite’s method (Kuznetsova *et al.*, 2017). One advantage of a mixed effect model is the ability to estimate fixed effects from incomplete data (West and Galecki, 2011). For the perfusion analysis, PETCO_2_ and age were demeaned and included as covariates in the model along with sex. *Post hoc* analyses examined Huntington’s disease and control participants separately to assess the effect of exercise.

Where a significant gene effect on CBF was found, a follow-up regression model was built from a set of *a priori* candidate predictor variables by entering and removing predictors based on the Akaike information criteria, in a stepwise manner. The *a priori* predictors entered were a genetic predictor (CAG repeat length), a clinical predictor (Unified Huntington’s disease Rating Scale total motor score), a performance-related predictor (average HRR achieved during the intervention) and a physiological predictor (PETCO_2_ change, known to modulate CBF).

For the cognitive tests with a motor component (Trail Making, speed and capacity of language processing, Symbol Digit Modalities Test), motor speed was included as a covariate in the analyses. Age was a covariate in all cognitive analyses.

To compare performance and the physiological and perceptual response during the exercise intervention, HR, lactate, ratings of perceived exertion, work rate and cycling speed were averaged across the intervention period, excluding the warm up and warm down periods, and compared between Huntington’s disease and control participants. All data are expressed as mean ± SEM unless stated.

### Data availability

The CBF imaging data, physiological data, data analysis scripts and R code used for statistical analysis will be made available upon request for academic and non-commercial purposes.

## Results

Huntington’s disease and control participants did not differ on gender, age, body mass index, fitness levels (measured using a VO_2_ peak test) and self-reported physical activity levels (all *P* > 0.05, Table 1). Unadjusted values are shown in Supplementary Table 1. The cerebrovascular reactivity data were not analysed due to insufficient statistical power (see Supplementary material).

### Physiological and self-reported response to the exercise intervention

There was no difference on any of the HR metrics between control and Huntington’s disease participants during the intervention; the exercise intervention caused a significant increase in HR compared to the upright rest period on the ergometer in both control and Huntington’s disease participants (exercise effect, *P* < 0.001). Control participants worked at an average 54.8 ± 6.1% of their maximum, calculated using HRR, compared to 54.8 ± 10.3% for Huntington’s disease participants (gene effect, *P* = 0.99). Average HR did not differ across the intervention for controls (126.8 ± 2.8 beats/min) and Huntington’s disease participants (127.2 ± 3.3 beats/min).

Mean lactate concentration across the intervention did not significantly differ between control participants (2.87 ± 0.22 mmol) and Huntington’s disease participants (2.23 ± 0.27 mmol; *P* = 0.07). Mean self-reported ratings of exertion were associated with the verbal anchor ‘moderate’ to ‘somewhat hard’ exertion on the Borg 0–10 rating of perceived exertion (RPE) scale for both controls and Huntington’s disease participants in the legs (controls = 3.89 ± 0.22; Huntington’s disease = 4.25 ± 0.50, *P* = 0.53) and for breathing (controls = 3.38 ± 0.21; Huntington’s disease = 3.44 ± 0.42, *P* = 0.89). Results across the time course of the intervention are shown in Supplementary Fig. 1.

Despite no differences in the physiological or self-reported response to the intervention, Huntington’s disease participants cycled at a significantly lower workload (60.93 ± 5.09 W) compared to controls (90.86 ± 4.77 W, *F*(1,35) = 18.29, *P* < 0.001, *η*^2^ = 0.34), whereas there was no group difference in cycling speed (controls = 69.67 ± 2.45 revolutions/min; Huntington’s disease participants = 71.63 ± 3.03 revolutions/min, *P* = 0.62).

Mean HR, mean arterial pressure and PETCO_2_ values for the baseline and post-exercise scan session are shown in Table 2. Exercise and gene did not interact on any measure (all *P* > 0.05). Exercise and gene status had a significant main effect on HR; Huntington’s disease participants’ HR was 7.77 ± 3.61 beats/min higher compared to controls (*P* = 0.037). After the exercise session, HR remained elevated across both groups at 15 min (+7.88 ± 1.23 beats/min, *P* = 5.23e−09) and 40 min post-exercise (+2.50 ± 1.18 beats/min, *P* = 0.037), before returning to baseline levels after 60 min (+1.46 ± 1.21 beats/min, *P* = 0.22).

**Table 2:** Estimated marginal means (±SEM) after adjusting for PETCO_2_, age and sex for cardiorespiratory and cerebrovascular measures at baseline and after 20 min of moderate-intensity exercise

	Baseline		Post-exercise scan session					
			Post-1		Post-2		Post-3	
	Controls	Huntington’s disease	Controls	Huntington’s disease	Controls	Huntington’s disease	Controls	Huntington’s disease
Cardiorespiratory measures								
HR (beats/min)	62.0 ± 2.2	69.8 ± 2.6	**69.9 ± 2.7**	**77.8 ± 2.5**	**65.2 ± 2.2**	72.2 ± 2.1	64.5 ± 2.6	74.2 ± 2.7
MAP (mmHg)	87.5 ± 2.2	92.7 ± 2.1	86.9 ± 2.5	90.2 ± 2.3	87.2 ± 3.0	89.9 ± 2.9	87.9 ± 2.8	92.5 ± 2.6
PETCO_2_ (mmHg)	36.2 ± 1.2	34.7 ± 1.4	**34.4 ± 1.2**	**31.6 ± 1.3**	**34.0 ± 1.2**	**30.3 ± 1.7**	**34.6 ± 1.1**	**32.0 ± 1.5**
Perfusion (ml/100 g/min) in ROIs								
Grey matter	52.8 ± 2.9	51.5 ± 3.2	53.3 ± 3.0	55.6 ± 3.2	50.2 ± 3.0	56.1 ± 3.4	52.0 ± 3.0	53.5 ± 3.4
Middle frontal gyrus	43.4 ± 4.2	38.8 ± 4.5	43.5 ± 4.4	41.5 ± 4.6	42.3 ± 4.4	**51.4 ± 5.1**	42.8 ± 4.6	39.1 ± 5.1
Postcentral gyrus	51.0 ± 4.7	48.5 ± 5.0	52.7 ± 4.8	54.9 ± 5.2	51.4 ± 4.8	60.9 ± 5.6	55.0 ± 4.9	54.1 ± 5.7
Precentral gyrus	54.4 ± 4.1	51.7 ± 4.4	52.8 ± 4.2	56.3 ± 4.5	50.2 ± 4.2	**61.6 ± 4.9**	51.5 ± 4.4	55.5 ± 4.9
Caudate	31.6 ± 3.1	24.3 ± 2.3	29.7 ± 2.3	26.1 ± 4.2	29.7 ± 2.9	26.1 ± 2.5	37.0 ± 6.8	25.7 ± 3.4
Thalamus	49.5 ± 4.8	41.1 ± 3.6	49.1 ± 3.4	42.8 ± 4.4	45.8 ± 4.4	46.3 ± 5.0	41.7 ± 3.1	40.7 ± 4.4
Hippocampus	51.0 ± 4.7	48.5 ± 5.0	52.7 ± 4.8	54.9 ± 5.2	51.4 ± 4.8	**60.9 ± 5.6**	55.0 ± 4.9	54.1 ± 5.7

Data in bold represent a significant effect of exercise in *post hoc* analyses.

MAP = mean arterial pressure; PETCO_2_ = end-tidal partial pressure of carbon dioxide.

Exercise and gene status had no effect on mean arterial pressure, with no evidence of exercise-induced hypotension when the post-exercise scan session began (*P* > 0.05).

**Figure 2:**
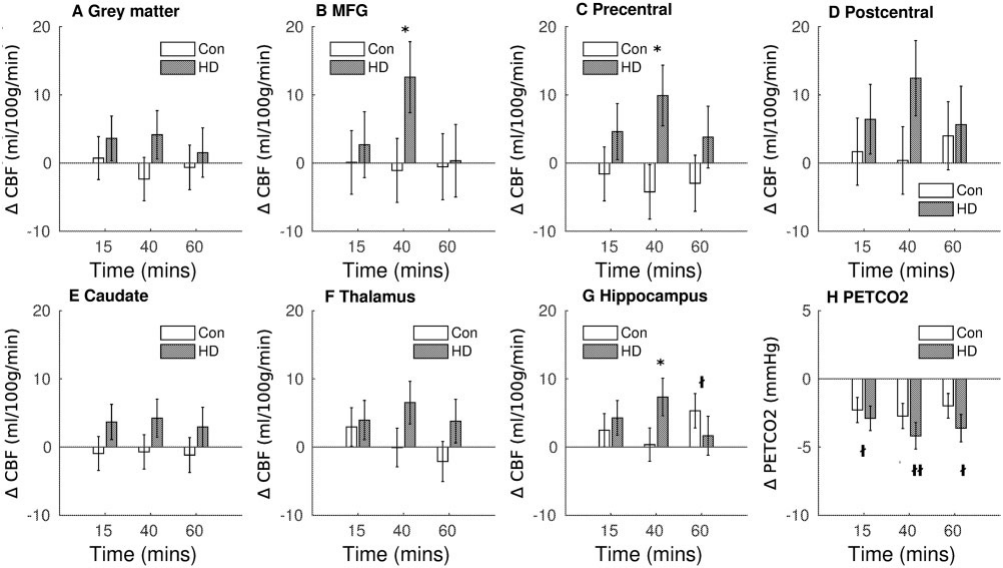
**Exercise-induced cerebral perfusion changes.** Absolute change in CBF and end-tidal CO_2_ (bottom right panel) from baseline, measured at 15, 40 and 60 min following exercise cessation. Data shown are the marginal means adjusted for PETCO_2_, sex and age. Error bars represent the SEM. CON = controls; MFG = middle frontal gyrus; PETCO_2_ = end-tidal partial pressure of carbon dioxide; *P < 0.05 gene effect; ^†^*P* < 0.05; ^††^*P* < 0.01 main effect of exercise.

PETCO_2_ levels were similar for Huntington’s disease and control participants, and exercise-induced hypocapnia was observed across both groups of participants and persisted for 60 min post-exercise, with a significant reduction in PETCO_2_ from baseline at 15 min (*t*_88.5_ = −2.48, *P* = 0.015), 40 min (*t*_88.5_ = −2.96, *P* = 0.004) and 60 min post-exercise (*t*_88.3_ = −2.19, *P* = 0.03) (see Fig. 2H).

### Regionally specific effects of exercise on blood flow in Huntington’s disease

Absolute CBF values (estimated marginal means adjusted for age, sex and end-tidal CO_2_) are reported in Table 2; the change in CBF from baseline is shown in Fig. 2 for the three post-exercise time points.

Globally, exercise (*P* = 0.80) and gene status (*P* = 0.73) had no effect on grey matter CBF. A gene status × exercise interaction was found in the MFG and precentral gyrus; 40 min after exercise cessation, the exercise-induced CBF change from baseline was 13.67 ± 6.75 ml/100 g/min higher in patients with Huntington’s disease in the MFG (*t*_81.8_ = 2.03, *P* = 0.046, Fig. 2B) and 14.12 ± 5.71 ml/100 g/min higher in the precentral gyrus in Huntington’s disease participants compared to controls (*t*_81.1_ = 2.47, *P* = 0.016, Fig. 2C). *Post hoc* analyses found that the change in CBF relative to baseline in the precentral and MFG was significant in the Huntington’s disease participants (precentral increase = 11.73 ± 4.36 ml/100 g/min, *P* = 0.010, MFG increase = 14.88 ± 5.65 ml/100 g/min, *P* = 0.012), whereas the change was not significant in control participants (precentral decrease = −4.61 ± 4.33, *P* = 0.29, MFG decrease = −2.25 ± 4.53, *P* = 0.62).

Controls and Huntington’s disease participants did not differ in precentral and MFG CBF at baseline (*P* = 0.60 and 0.32, respectively), 15-min post-exercise (6.20 ± 5.52 and 2.60 ± 6.54 ml/100 g/min respectively) and 60-min post-exercise (6.77 ± 5.96 and 0.89 ± 7.03 ml/100 g/min; all *P* > 0.05). Exercise and gene status had no effect on CBF values in the postcentral gyrus.

In the subcortical ROIs, hippocampal CBF was 5.12 ± 2.45 ml/100 g/min higher 60 min post-exercise across all participants (*t*_205_ = 2.07, *P* = 0.039). In addition, an interaction between exercise and gene status was found (*F*(3,198) = 3.05, *PP* = 0.03); at 40 min post-exercise, the exercise-induced CBF change was significantly different between Huntington’s disease and control participants (6.98 ± 3.51 ml/100 g/min; *t*_200_ = 1.99, *P* = 0.048, see Fig. 2G). *Post hoc* analysis found that this was driven by a significant increase in hippocampal CBF in Huntington’s disease participants 40 min after exercise cessation (+6.53 ± 3.15 ml/100 g/min, *P* = 0.04), whereas there was no significant change in control participants at this time (1.28 ± 2.30, *P* = 0.58). Baseline hippocampal CBF did not differ between controls and Huntington’s disease participants (*t*_68_ = 0.12, *P* = 0.90).

Exercise and gene status had no effect on CBF in the thalamus or caudate and no effect on arterial arrival time averaged across the grey matter or in any of the ROIs (all *P* > 0.05, Supplementary Table 1).

### Predictors of the exercise-induced CBF response

VO_2_ peak was not correlated with baseline CBF in grey matter or in the ROIs, and gene status did not affect the relationship (all *P* > 0.05).

**Figure 3:**
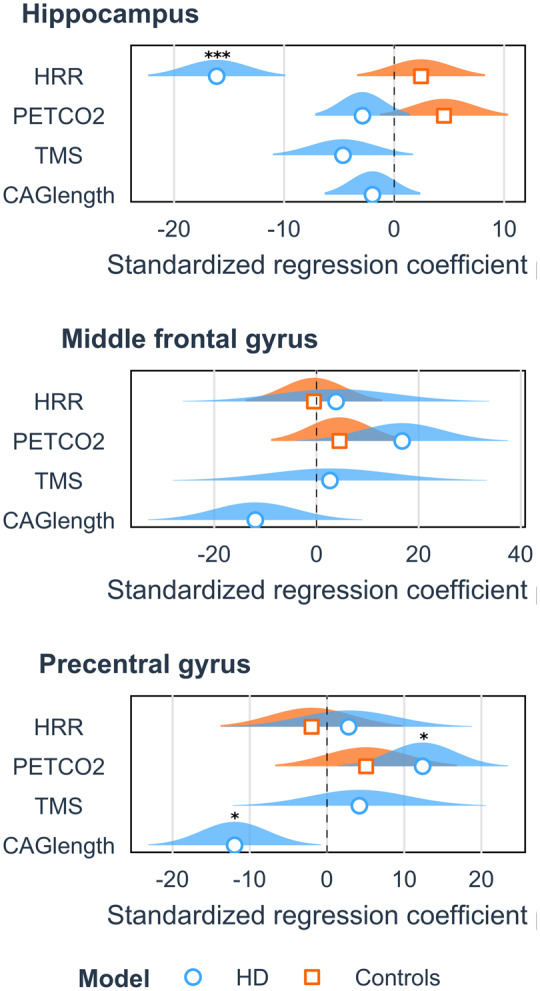
**Predictors of cerebral perfusion change.** Statistics from linear model at 40 min after exercise cessation. Scaled coefficient estimates, 95% confidence intervals and coefficient of uncertainty shown. *P < 0.05; ***P < 0.001. HRR = heart rate reserve; TMS = total motor score.

A regression model examined the predictors of the observed perfusion changes seen 40 min after exercise cessation; the scaled estimates for each predictor in the model for Huntington’s disease and control participants are shown in Fig. 3.

In Huntington’s disease participants, HRR, PETCO_2_ change and total motor score were entered into the regression model and explained 68.2% of the variance in exercise-induced CBF change in the hippocampus (*P* = 0.0001). HRR explained 42.8% of the variance alone (*β* = −148.8, *P* < 0.001), whilst PETCO_2_ and total motor score were non-significant predictors (*β* = −0.62 and −0.35, *P* = 0.129 and 0.086, respectively).

In the precentral gyrus, 57% of the variance in CBF change was explained by the model that included CAG repeat length (*β* = −4.17, *P* = 0.031), PETCO_2_ change (*β* = 2.38, *P* = 0.022) and HRR during the intervention (*β* = −1.62, *P* = 0.97). The clinical predictor (total motor score) did not meet the criteria for entry into the model.

In the MFG, PETCO_2_ change (*β* = 3.20, *P* = 0.06), CAG repeat length (*β* = −4.26, *P* = 0.16) and HRR (*β* = 17.26, *P* = 0.80) explained 29.1% of the CBF variance and the model was not significant (*P* = 0.18).

In control participants at the same time point 40 min post-exercise cessation, HRR and PETCO_2_ were not predictive of hippocampal CBF change (adjusted *R*^2^ = 0.05, *P* = 0.20), MFG CBF change (adjusted *R*^2^ = −0.13, *P* = 0.76) or precentral CBF change (adjusted *R*^2^ = −0.08, *P* = 0.62).

### Cognitive performance is impaired following acute exercise

Cognitive test performance is shown in Table 3; Huntington’s disease participants performed worse on all cognitive tests (main effect of gene status, all *P* < 0.05) and had a significantly slower motor tapping speed compared to controls (*F*(1,33) = 17.06, *P* = 0.0002).

**Table 3:** Cognitive performance at baseline and 75-min after exercise cessation

	Controls		Huntington’s disease		FDR-adjusted *P*-values	
	Baseline	Post-exercise	Baseline	Post-exercise	Exercise	Gene status
Symbol digit score	61.3 ± 2.9	57.2 ± 3.0	41.8 ± 3.1	40.4 ± 3.2	**0.015**	**0.0004**
SCOLP	84.5 ± 4.8	78.6 ± 4.9	52.1 ± 5.1	50.1 ± 5.2	**0.012**	**0.0004**
Digit span (raw)	11.7 ± 0.5	11.8 ± 0.6	9.7 ± 0.6	9.1 ± 0.6	0.3	**0.004**
Trail making (s)	44.7 ± 11.7	51.3 ± 12.2	96.9 ± 11.8	106.8 ± 13.1	0.14	**0.0053**
Verbal fluency (1 letter)	17.8 ± 1.2	15.5 ± 1.2	9.7 ± 1.2	10.3 ± 1.2	0.26	**0.0004**
Stroop interference	50.1 ± 3.1	44.8 ± 3.1	36.0 ± 3.13	33.2 ± 3.1	**0.012**	**0.005**
Motor speed (taps/min)	452 ± 20.9	459 ± 21.3	337 ± 21.4	331 ± 21.3	0.96	**0.0004**

Mean ± SEM. Data are marginal means adjusted for age. For the symbol digit score, SCOLP, and trail making, data are also adjusted for motor speed. Data in bold represent a significant effect q < 0.05.

SCOLP = speed and capacity of language processing.

A main effect of exercise was found for performance on the Stroop interference task (*P* = 0.002), symbol digits modality test (*P* = 0.007) and speed and capacity of language processing test (*P* = 0.003), with worse performance observed following the exercise intervention compared to baseline (see Table 3). There was no interaction between the effect of exercise and gene status.

## Discussion

Long-term exercise interventions improve motor functioning in people with Huntington’s disease (Khalil *et al.*, 2013; Quinn *et al.*, 2016; Fritz *et al.*, 2017), although the underlying mechanism is poorly understood. Here, using a highly controlled acute exercise paradigm, we aimed to characterize the cerebrovascular response to a single session of aerobic exercise in people with Huntington’s disease using ASL MRI. Our data showed that 20 min of moderate-intensity cycling induced a transient regionally selective cerebral perfusion response that was different in Huntington’s disease participants compared to control participants. Whilst exercise induced a non-significant negative change in CBF in control participants in the precentral and middle frontal gyri, a significant increase in CBF was observed in Huntington’s disease participants 40 min after exercise cessation. Likewise, in the hippocampus, an area previously shown to be selectively responsive to acute exercise (Steventon *et al.*, 2020), CBF was significantly elevated 40 min after exercise in Huntington’s disease participants compared to controls. We further observed an increase in hippocampal blood flow 60 min after exercise cessation in both controls and Huntington’s disease participants, in line with previous work (Steventon *et al.*, 2020).

The differential effect of exercise on cerebral perfusion in Huntington’s disease participants compared to controls was hypothesized based on an altered cardiovascular response to exercise (Steventon *et al.*, 2018) and resting cerebrovascular abnormalities (Drouin-Ouellet *et al.*, 2015); however, the direction of perfusion effects in Huntington’s disease participants was not anticipated, with an increase in CBF generally interpreted as beneficial in healthy cohorts. However, as an increase in CBF was not also seen in controls, the cerebrovascular response more likely reflects a latent pathology induced by exercise in Huntington’s disease participants. In support of this, cerebral hyper-perfusion has been documented previously in patients with Huntington’s disease and animal models, observed as increased vessel density, increased cerebral blood volume and flow, increased blood brain barrier permeability and greater release of VEGF-A, an angiogenic growth factor, by astrocytes (Vis *et al.*, 1998; Harris *et al.*, 1999; Wolf *et al.*, 2011; Chen *et al.*, 2012; Franciosi *et al.*, 2012; Lin *et al.*, 2013; Hua *et al.*, 2014; Hsiao *et al.*, 2015). Increased angiogenesis coupled with a reduced number of pericytes and altered vascular reactivity has been observed in Huntington’s disease mice (Hsiao *et al.*, 2015), suggesting a complex functional impairment that may impact neurovascular coupling and thus the cerebrovascular response to exercise.

The regional specificity of the exercise effects is particularly noteworthy and partly in agreement with previous work in young adults and patients with stroke (MacIntosh *et al.*, 2014; Robertson *et al.*, 2015; Steventon *et al.*, 2020), suggesting that the observed effects are specific to the exercise intervention in the Huntington’s disease group. Moreover, CAG repeat length, a key factor in Huntington’s disease pathogenesis, was predictive of the cerebrovascular response to exercise in the precentral gyrus, with a larger post-exercise increase in CBF observed in participants with a lower CAG repeat length. The precentral gyrus, also known as the primary motor cortex, is a vital structure involved in executing voluntary motor movements and capable of cortical functional reorganization (Wall *et al.*, 2002; Lee *et al.*, 2003) and acute compensatory plasticity, with changes in functional organization demonstrated over minutes, weeks and longer durations (Wall *et al.*, 2002; Lee *et al.*, 2003; Weiss *et al.*, 2004; Björkman *et al.*, 2009). The regional effects were not explained by baseline resting hypo- or hyper-perfusion in the Huntington’s disease participants; however, previous work has shown significant cortical thinning and locally decreased task-related fMRI activation in both the middle frontal and precentral gyri in Huntington’s disease participants (Wolf *et al.*, 2007, 2008; Rosas *et al.*, 2008; Saft *et al.*, 2008). This supports the concept that the regionally selective effect in Huntington’s disease participants and not controls are due to underlying vascular alterations in Huntington’s disease, which convey an increased propensity for exercise-induced vascular adaptions. However, increased connectivity has been observed using resting-state fMRI in the precentral gyrus in pre-manifest Huntington’s disease. This could alternatively suggest that regional compensatory changes occur in brain connectivity early on in Huntington’s disease (Koenig *et al.*, 2014), and the observed relationship observed with CAG length suggesting the capacity for compensatory changes is dependent on genetic burden. Further research is warranted to test whether the observed increase in CBF could reflect a compensatory mechanism to support neuronal survival, as certain regions may be less vulnerable to hindered cerebral haemodynamics in individuals with a lower genetic load.

The regulation of CBF is controlled by neurogenic, metabolic, autoregulatory and systemic factors; exercise-induced changes in CBF may be accounted for by changes in some or all of these in Huntington’s disease participants. However, the temporal pattern of cardiorespiratory recovery after exercise cannot explain our results; HR, blood pressure and the PETCO_2_ were not differentially affected by exercise in Huntington’s disease and control participants. In both groups, blood pressure recovered to baseline levels prior to the post-exercise scans and HR was elevated during both the 15- and 40-min post-exercise scan, whereas a CBF difference was only observed at 40 min. PETCO_2_ remained significantly lower than baseline at all three post-exercise time points, and because PETCO_2_ is known to modulate arteriolar diameter (Thomas *et al.*, 1989; Ide and Secher, 2000; Ratanakorn *et al.*, 2001), it was accounted for in the statistical model and predicted the CBF change in the precentral gyrus in Huntington’s disease participants. However, this is unlikely to explain the differences seen between controls and Huntington’s disease participants; the magnitude of PETCO_2_ change did not differ between the two groups and PETCO_2_ did not account for the changes observed in the hippocampus and MFG. In a healthy vascular system, hypocapnia (observed here following exercise) causes an increase in cerebral vasoconstriction; therefore, a reduction in CBF would be expected, rather than the observed increase in CBF seen in Huntington’s disease participants. However, impaired vascular reactivity to haemodynamic challenges has been shown in preclinical Huntington’s disease models, with a smaller increase in CBF following carbogen in Huntington’s disease mice compared to wild types, despite greater vessel density (Hsiao *et al.*, 2015). Thus, the observed increase in CBF after exercise in patients with Huntington’s disease may be due to a blunted hypocapnic response in Huntington’s disease participants, although the results from the regression model do not support this in all of the regions affected. We intended to measure the CBF response to hypercapnia in this study to test this hypothesis; however, the breath-hold paradigm we utilized was limited by highly variable and poor task performance in both groups and, thus, requires further optimization before a robust measure of cerebrovascular reactivity can be obtained.

The temporal specificity of our results is particularly novel, with a difference between Huntington’s disease and controls observed 40 min after exercise cessation, but not at 15 and 60 min post-exercise. One explanation for the CBF difference is a lag effect from the CVR breath-hold challenge, performed immediately before the 40-min CBF scan, as during breath-holding, the increase in the partial pressure of CO_2_ gives rise to increased CBF because of vasomotor reactivity. However, this is unlikely due to poor performance in both groups, with CO_2_ increasing after a breath-hold on only half of the trials. More likely, it suggests that there is a temporal window for acute exercise effects that may be driven by resting vascular perturbations in the Huntington’s disease participants. In support of this, work in patients with stroke also shows a transient time-dependent CBF response to exercise in distinct brain regions, with a reduction in CBF in the MFG observed 30-min post-exercise, and returning to baseline by 50 min (Robertson *et al.*, 2015).

Designing an exercise intervention to be physically and perceptually similar for the patient and control group was challenging given that patients with Huntington’s disease have an altered metabolic and cardiorespiratory response to submaximal exercise (Steventon *et al.*, 2018). Nevertheless, we achieved an equivalent moderate-intensity aerobic intervention with no difference in the peripheral physiological response or self-reported exertion, suggesting that the observed effects in Huntington’s disease are not due to a difference in intervention prescription. However, Huntington’s disease participants on average cycled at a lower workload to achieve the prescribed target HR, most likely due to an altered movement economy. The exercise intensity achieved by Huntington’s disease participants during the intervention predicted the hippocampal perfusion response, which may indicate that unlike the cortical regions, the exercise-induced hippocampal effect is intensity dependent. It is plausible that cardiovascular changes observed in preclinical models and clinical Huntington’s disease (Wood *et al.*, 2012; Buonincontri *et al.*, 2014; Bellosta Diago *et al.*, 2018; Steventon *et al.*, 2018) may mediate the altered cerebral response observed. Huntington’s disease participants were found to have a higher HR across the study compared to controls, although no interaction effect was found with exercise. Future studies would combine comprehensive cardiovascular and cerebrovascular measures to investigate vascular impairments in Huntington’s disease in response to exercise.

An additional consideration is that exercise is a potent physiological stimulus upon the hypothalamo-pituitary adrenal axis. There is evidence that the hypothalamo-pituitary adrenal axis is impaired in Huntington’s disease (Kassubek *et al.*, 2004; Petersén *et al.*, 2005; Björkqvist *et al.*, 2006; Soneson *et al.*, 2010; van Wamelen *et al.*, 2014), with mixed evidence of subtle alterations in neuroendocrine signalling, including cortisol (Aziz *et al.*, 2009; Kalliolia *et al.*, 2015). Equivocal evidence for a modulating effect of cortisol on CBF (Hodkinson *et al.*, 2014; Schilling *et al.*, 2014; Handley *et al.*, 2016) is sufficient to merit future exercise studies in Huntington’s disease collecting neuroendocrine measures along with cardiorespiratory data.

Functional improvements in cognition have been reported following long-term exercise interventions in healthy cohorts (Dustman *et al.*, 1984; Kramer *et al.*, 1999) as well as in Parkinson’s disease (Duchesne *et al.*, 2015; Altmann *et al.*, 2016) and Alzheimer’s disease (Ströhle *et al.*, 2015; Teixeira *et al.*, 2018). In this study, cognitive performance 1-h after exercise cessation was worse than at baseline in Huntington’s disease and control participants alike, most likely due to fatigue associated with the lengthy testing session, rather than a direct effect of exercise. Whereas baseline cognitive tests were completed at the beginning of the experimental session on a separate day, the post-exercise testing was conducted 3.5 h into the experimental session. Cognitive data were a secondary outcome measure; it is plausible that transient cognitive gains returned to baseline levels by the time of testing, with the study not optimally designed to examine transient cognitive effects and appropriately control for fatigue. We were also likely underpowered to detect an effect, with acute gains in cognition previously reported with small effect sizes (Chang *et al.*, 2012).

A limitation of our study is the heterogeneity of the Huntington’s disease group, which includes pre-symptomatic and manifests gene carriers. Therefore, we were unable to identify at what stage in the disease an altered response to exercise occurs. Future work in a pre-symptomatic group is required to provide insight into whether the observed exercise-induced brain changes are causally involved in early pathogenesis of Huntington’s disease.

## Conclusions

Overall, we observed a differential response to a single session of exercise in Huntington’s disease and control participants, with a transient regionally selective increase in perfusion in Huntington’s disease participants. The highly controlled acute exercise paradigm used may provide a framework for determining the key components that enable exercise to modulate a pathologically disturbed cerebrovasculature for therapeutic gain in Huntington’s disease. Further work is necessary to understand the extent and pattern of disruptions to the neurovascular unit in Huntington’s disease to inform the development of targeted exercise approaches.

## Supplementary material

Supplementary material is available at *Brain Communications* online.

## Supplementary Material

fcaa044_Supplementary_Data

## Abbreviations

ASL: = arterial-spin labelling
CAG: = cytosine–adenine–guanine
CBF: = cerebral blood flow
HR: = heart rate
HRR: = heart rate reserve
MFG: = middle frontal gyrus
PETCO_2_: = end-tidal partial pressure of carbon dioxide
ROI: = region of interest
