# Supplementary material for: Altered cerebrovascular response to acute exercise in patients with Huntington’s disease
Source: Brain Commun. 2020 Apr 16;2(1):fcaa044. doi: 10.1093/braincomms/fcaa044 (PMC7293798; doi:10.1093/braincomms/fcaa044)
Supplement: fcaa044_Supplementary_Data [file fcaa044_supplementary_data.zip › Original_submission.pdf]

OXFORD UNIVERSITY PRESS | Brain Communications

Altered cerebrovascular response to acute exercise in patients with Huntington's Disease

Journal:	*Brain Communications*
Manuscript ID	BRAINCOM-2019-192
Manuscript Type:	Original Article
Date Submitted by the Author:	17-Dec-2019
Complete List of Authors:	Steventon, Jessica; Cardiff University, CUBRIC, School of Physics and Astronomy<br>Furby, Hannah; Cardiff University, Neuroscience and Mental Health Research Institute; Cardiff University, 3. Cardiff University Brain Research Imaging Centre, School of Psychology, Maindy Road, Cardiff University, UK. CF24 4HQ<br>Ralph, James; Cardiff University, CUBRIC, School of Psychology<br>O'Callaghan, Peter; University Hospital of Wales Healthcare NHS Trust, Cardiology Department<br>Rosser, Anne; Cardiff University, Neuroscience and Mental Health Research Institute; Cardiff University, Cardiff Brain Repair Group, School of Biosciences,<br>Wise, Richard; University of Cardiff, CUBRIC, School of Psychology<br>Busse, M E; Cardiff University, Centre for Trials Research<br>Murphy, Kevin; Cardiff University, CUBRIC, School of Physics and Astronomy
Keywords:	cerebral blood flow, neurodegeneration, plasticity

SCHOLARONE™
Manuscripts

Altered cerebrovascular response to acute exercise in patients with Huntington's Disease

Authors and affiliations

J. J. Steventon[1,2] H. Furby[2,3], J.Ralph[3], P. O'Callaghan[4], A.E.Rosser[2,6], R.Wise[3], M. Busse[5], K.Murphy[1]

1. Cardiff University Brain Research Imaging Centre, School of Physics and Astronomy, Cardiff University, UK. CF24 4HQ
2. Neuroscience and Mental Health Research Institute, School of Medicine, Maindy Road, Cardiff, UK. CF24 4HQ
3. Cardiff University Brain Research Imaging Centre, School of Psychology, Maindy Road, Cardiff University, UK. CF24 4HQ
4. Cardiology Department, University Hospital of Wales, Cardiff, UK
5. Centre for Trials Research, Cardiff University, UK
6. Cardiff Brain Repair Group, School of Biosciences, Museum Avenue, Cardiff University, UK. CF10 3AX

Corresponding Author: *Jessica J Steventon,* Cardiff University Brain Research Imaging Centre, Maindy Road, Cardiff University, UK. CF24 4HQ *Phone: +44 (0) 29 206 88758. Email:* steventonjj@cardiff.ac.uk

Running title: Exercise MRI in Huntington's Disease

Keywords: neurodegeneration, plasticity, cerebral blood flow, MRI

Word count : 5,585

For Review Only

Abstract

Objective. To determine whether a single session of exercise is sufficient to induce cerebral adaptations in individuals with Huntington's disease, and explore the time dynamics of any acute cerebrovascular response.

Methods. In this case-control study we employed arterial-spin labelling magnetic resonance imaging in 19 HD gene-positive participants (32-65 years old, 13 males) and 19 controls (29-63 years old, 10 males) matched for age, gender, body mass index and self-reported activity levels, to measure global and regional perfusion in response to 20-minutes of moderate-intensity cycling. Cerebral perfusion was measured at baseline and 15-, 40- and 60-minutes after exercise cessation.

Results. Relative to baseline, cerebral perfusion increased in HD patients and decreased in control participants in the precentral gyrus, middle frontal gyrus and hippocampus 40-minutes after exercise cessation (+15 to +32.5% change in HD participants, -7.7 to 0.8% change in controls). CAG repeat length predicted the change in the precentral gyrus, and the intensity of the exercise intervention predicted hippocampal perfusion change in HD participants. In both groups, exercise increased hippocampal blood flow 60-minutes after exercise cessation.

Conclusions. Here we demonstrate the utility of acute exercise as a clinically sensitive experimental paradigm to modulate the cerebrovasculature. Twenty minutes of aerobic exercise induced transient cerebrovascular adaptations in the hippocampus and cortex selectively in HD participants and likely represents latent neuropathology not evident at rest.

For Review Only

Introduction

Huntington's disease (HD) is a progressive neurodegenerative disorder caused by the expansion of a polyglutamine stretch within the Huntingtin gene(Gusella *et al.*, 1993; Vonsattel and DiFiglia, 1998). Neuropathology in HD causes cognitive dysfunction, psychiatric symptoms, and a movement disorder characterized by involuntary movements and impaired motor control(Bates *et al.*, 2002). The availability of genetic testing means at-risk HD family members can be identified early in the disease course and prior to the onset of symptoms. As a result, there is a drive to discover the earliest signs of neuropathology that can guide future therapeutics.

Current evidence suggests that long-term exercise interventions can produce functional improvements in a range of populations. Exercise studies using genetic mouse models of HD have produced promising results, with converging evidence showing an improvement, or delay, in the emergence of motor impairments (van Dellen *et al.*, 2000, 2008; Pang *et al.*, 2006; Harrison *et al.*, 2013; Herbst and Holloway, 2015). Similarly a systematic review of 18 patient studies found evidence for beneficial effects of exercise on motor function, gait speed and balance in participants with HD(Fritz *et al.*, 2017). Despite this, the mechanisms supporting these exercise-induced functional improvements in HD are unclear.

Evidence for a beneficial effect of exercise on HD neuropathology is largely limited to genetic rodent models of HD and remains equivocal. A reduction in striatal neuropathology has been observed by some studies(Pang *et al.*, 2006; Harrison *et al.*, 2013), whilst others have shown no effect of exercise on the degree of atrophy or mHtt aggregation(van Dellen *et al.*, 2008). In a small patient study, a 9-month multi-disciplinary rehabilitation intervention which included exercise as a component was found to increase

brain volume in the grey matter, caudate and prefrontal cortex and improve cognition, although the independent contribution of exercise was not determined(Cruickshank *et al.*, 2015).

Inconsistencies across studies in the type, dose and duration of the exercise programme are likely to account for the discrepancies in the literature and may be masking the therapeutic potential of exercise in HD. To address this, a focused investigation of the temporal dynamics of exercise is needed. Emerging evidence suggests regional exercise effects on the brain may overlap in terms of acute and long-term timescales(Weng *et al.*, 2017). For example, in healthy adults, a single exercise session has been shown to improve motor function(Mang *et al.*, 2014; Skriver *et al.*, 2014) improve cognition(Winter *et al.*, 2007; Coles and Tomporowski, 2008, Chang *et al.*, 2012*a*; Roig *et al.*, 2012; Jo *et al.*, 2018), increase peripheral neurotrophin and catecholamine biomarkers(Winter *et al.*, 2007; Mang *et al.*, 2014; Skriver *et al.*, 2014) and alter cerebral blood flow(Smith *et al.*, 2010; MacIntosh *et al.*, 2014; Steventon *et al.*, 2019). However, it is not clear whether the cerebrovascular response to the physiological provocation of exercise will be the same in a disease group where resting perturbations in the vascular system are present. In HD, structural, morphological and functional cerebral vascular abnormalities have been shown(Vis *et al.*, 1998; Lin *et al.*, 2013; Rahman *et al.*, 2013; Hua *et al.*, 2014; Drouin-Ouellet *et al.*, 2015; Hsiao *et al.*, 2015; St-Amour *et al.*, 2015) and aggregation of mutant Huntingtin is present in the neurovascular unit(Drouin-Ouellet *et al.*, 2015) which is likely to affect the ability of the vascular system to respond to the demands of acute exercise.

The current study is the first to examine the cerebrovascular response to a single session of exercise in Huntington's Disease. Previous work has shown an altered peripheral response during and following exercise(Steventon *et al.*, 2018), with metabolic and cardiorespiratory deficits found to reduce exercise performance and affect exercise recovery during an incremental exercise test.

Arterial spin labelling (ASL) MRI is a quantitative, non-invasive method to measure tissue perfusion and uses magnetically labelled arterial blood water protons as an endogenous tracer. ASL MRI has been shown to be a reliable and repeatable method for quantifying tissue perfusion in aged and clinical populations(Kilroy *et al.*, 2014), and a single session of exercise has been shown to produce transient perfusion changes detectable with ASL MRI in healthy populations(Smith *et al.*, 2010; MacIntosh *et al.*, 2014; Steventon *et al.*, 2019). In stroke patients – a patient cohort where baseline cerebrovascular perturbations are evident - 20-minutes of low and moderate intensity cycling resulted in an intensity-dependent change in perfusion in the somatosensory cortex using ASL-MRI, along with an intensity-independent response in the basal ganglia(Robertson *et al.*, 2015).

Here we examined the effect of exercise on the cerebrovasculature in order to establish if the acute response to a single session of exercise is different in HD. Our primary hypothesis was a differential effect of exercise on CBF in HD patients compared to controls, based on evidence of cerebrovascular abnormalities in HD(Drouin-Ouellet *et al.*, 2015) and an altered cardiorespiratory response to submaximal exercise(Steventon *et al.*, 2018) .

review Only

Methods

The study design is detailed in Figure 1.

Participants

HD gene-positive participants (n = 19) were recruited from the South Wales HD research and management clinic, based in Cardiff, along with19 healthy age matched controls. Gene carriers included participants in the pre-manifest (N=7) and manifest stages of disease (Stage I N=3; Stage II N=6; Stage III N=3) according to their total functional capacity (TFC) on the Unified Huntington's Disease Rating Scale (UHDRS) as defined previously(Shoulson and Fahn, 1979).

Demographic and clinical data are shown in **Error! Reference source not found.**. All participants had a stable medication regime, defined as unchanged for four weeks. Exclusion criteria included any physical or psychiatric condition that would prohibit the participant from completing the exercise test, the inability to independently use the cycle ergometer, the inability to follow the protocol instructions, uncontrolled arterial hypertension, any neurological condition other than HD, pregnancy or childbirth in the last 6 weeks, current and/or history of cardiac, vascular or respiratory/pulmonary conditions, illicit drug use in the last four weeks and any MRI contraindications. Data was collected with ethical approval from Wales Research Ethics Committee (15/WA/0074) and all participants gave informed consent according to the Declaration of Helsinki. All HD gene carriers were registered on a global longitudinal study of HD patients (ENROLL-HD; REC no. 04/WSE05/89) and had been examined on the Unified Huntington's Disease Rating Scale (UHDRS) in the 6-month period prior to scanning thus the clinical and genetic data were available.

Exercise Intervention

The exercise intervention followed the procedures as described previously (Steventon *et al.*, 2019). Participants underwent 20-minutes of moderate-intensity aerobic cycling on an upright ergometer (Lode Ergometer, Lode, Groningen, Netherlands) at a prescribed intensity of 50-70% of the maximal heart rate reserve (HRR) determined using the Karvonen formula(American College of Sports Medicine. *et al.*, 2012).

During upright rest, the warm up and warm down period, and at three-minute intervals during exercise, lactate concentration was collected, blood pressure was measured and self-report ratings of perceived exertion were recorded using a 10-point Borg scale(Borg, 1974). After 20-minutes, participants completed a 2-minute warm down and then immediately returned to the MR scanner.

For Review Only

MRI acquisition and image processing

MRI of the brain was performed on a 3 T whole-body MRI system (GE Excite HDx, Milwaukee, WI, USA) equipped with a body transmit and eight-channel receive head coil.

For the quantitative measurement of cerebral perfusion (ml/100g/min) at baseline and at three timepoints post exercise (see Figure 1), a PICORE Pulsed Arterial Spin Labeling (PASL) sequence was performed(Wong *et al.*, 1997) with a gradient-echo spiral readout at eight inversion times ($TI_{1\text{-}8}$ = 400, 500, 600, 700, 1100, 1400, 1700, 2000ms, two separate scan series, echo time (TE) = 2.7 ms, TR = variable, 15 slices [7 mm thick + 1.5 mm gap], slice delay = 52 ms, FOV = 198 mm, 64 × 64 matrix = ~ 3.1 mm^2 in-plane resolution, 20cm tag width). A Quantitative Imaging of Perfusion with a Single Subtraction (QUIPSSII)(Wong *et al.*, 1998) cut-off of the label was applied at 700 ms for TIs > 700 ms. To estimate the equilibrium magnetization (M_0) of arterial blood, a single echo spiral k-space scan was acquired with the same parameters as above, minus the ASL tag preparation. A minimum contrast scan (TE/TR = 11/2000 ms) was also acquired to correct for field inhomogeneity(Wu *et al.*, 2011).

A 3-dimensional T_1-weighted fast spoiled gradient echo sequence was acquired at baseline and in the post-exercise scan session for image registration purposes (256 x 256, slice thickness = 1 mm giving a resolution of 1mm^3, TR/TE of 7.90/3.0 ms).

At baseline and 20-minutes post exercise, a breath-hold challenge was performed to measure cerebrovascular reactivity (10 end-expiration breath-holds of 15s length interleaved with 30s periods of paced breathing) with a single-shot PICORE QUIPSS II pulse sequence(Wong *et al.*, 1998). However, due to difficulties following the task instructions and poor performance of the breath-holds during acquisition, this data was not analyzed (see Supplementary Data).

Cerebral blood flow (CBF) quantification followed the procedures described by Steventon et al. (2019). Data were analysed in AFNI(Cox, 1996) and FSL

For Review Only

(http://fsl.fmrib.ox.ac.uk)(Jenkinson *et al.*, 2012). Images were motion corrected, brain extracted and an $M0_{CSF}$ image was created following the procedures described by Warnert et al. (2015). The M0 for arterial blood was then calculated according to methods described by Wong et al. (1998) with CSF as a reference. Perfusion quantification was performed on a voxel-by-voxel basis using a two-compartment model(Chappell *et al.*, 2010) and employing partial volume correction(Chappell *et al.*, 2011) to address signal contamination associated with atrophy.

Median grey matter values were calculated for the four perfusion images (baseline, post-1, post-2, post-3) to assess global perfusion. Based on the exercise literature(Pereira *et al.*, 2007; Smith *et al.*, 2010; MacIntosh *et al.*, 2014), regional changes in perfusion were assessed in a number of a priori defined regions of interest (ROIs): the thalamus (a key hub for the motor system), the hippocampus (evidence for exercise-induced neurogenesis(van Praag *et al.*, 2005; Pereira *et al.*, 2007)), and three key cortical ROIs involved in sensorimotor processing - the middle frontal gyrus, postcentral gyrus, and precentral gyrus. Perfusion was also examined in the caudate, based on the hallmark striatal pathology in HD(Paulsen *et al.*, 2010).

ROIs were segmented from the T_1-weighted image acquired in the same scan session and the median CBF values and arterial arrival time (AAT) - the time taken for blood to travel from the labelling slab to the tissue(Wang *et al.*, 2003; Zappe *et al.*, 2007), were calculated for each ROI.

Cardiorespiratory measures

Pulse waveforms and oxygen saturation were recorded (Medrad, PA, USA) and blood pressure measurements were collected using an arm-cuff before and after each scan (OMRON, Tokyo, Japan). Expired gas content was recorded (AEI Technologies, PA, USA) and sampled at 500 Hz (CED, Cambridge, UK) to obtain measures of $P_{ET}CO_2$. A respiratory belt was placed just below the ribs to monitor ventilation. Mean arterial pressure (MAP) was calculated as

For Review Only

(systolic + 2* diastolic)/3. Supine resting heart rate and blood pressure were measured after 15-minutes rest.

Neuropsychological testing

To capture any functional cognitive gains, participants were invited to complete 'baseline' cognitive tests on a separate day within 1 month of completing the MRI session, before or after to control for order effects. The post-exercise tests were completed immediately after the second MRI scan, at approximately 60-90 minutes after exercise cessation.

Five pencil and paper tasks were used to measure cognitive functioning: [1] The Forward Digit Span (WAIS-IV) was used as a measure of verbal short-term memory. Participants were asked to repeat a digit sequence orally, beginning with two digits, and increasing in length by one digit following successful repetition of two lists of digits at a given length. [2] The letter verbal fluency test was used to assess verbal functioning. Participants were required to produce words beginning with a certain letter of the alphabet in three respective 60-s trials(Lezak, 2012). [3] The Trail Making Test (part B) was used to measure visual attention, speed of processing, mental flexibility, and executive functions. Participants were presented with stimuli consisting of randomly placed numbers and letters and were required to connect the numbers of letters in sequence and alternating order (e.g. 1-A-2-B). [4] The Symbol Digit Modalities Test was used as a motor and psychomotor speed, with participants required to identify as many numbers [1-9] corresponding to a set of symbols in a single 90-s. [5] The Speed of Comprehension subtest from the Speed and Capacity of Language Processing (SCOLP) test was used as a measure of processing speed; participants were required to determine if a list of sentences were correct or incorrect within a 2-minute period. As motor speed may confound performance on many of the cognitive tests, participants completed a computer-based Speeded Tapping Test(Reitan, 1979). Participants were instructed to form a fist shape with their dominant hand and extend their

For Review Only

index finger to tap the space bar as quickly as possible. After 20 taps, the average number of taps per minute was calculated with performance averaged over three trials.

Clinical and genetic predictor measures

The motor component (total motor score; TMS) of the UHDRS provides a summed score of motor function including chorea, dystonia, motor impersistence, gait, rigidity, bradykinesia, finger tapping, and ocular movements. CAG repeat length was also used as a predictor.

Baseline fitness test

To measure baseline fitness, participants underwent an incremental cycle ergometer exercise test (1000W Cranlea, Human Performance Ltd, Birmingham, UK) on a separate day as described previously(Steventon *et al.*, 2018). The test protocol consisted of 2 minutes of rest, 2 minutes of unloaded cycling, followed by 25-watt increments every 2 minutes, starting at 50-watts. The exercise test was symptom limited; individuals were instructed to pedal until discomfort or fatigue prevented them from maintaining the required cadence. Pulmonary gas exchange was measured on a breath-by-breath basis (MetaMax 3B, Cortex Biophysik GmbH, Leipzig, Germany), with the gas analyser calibrated before each session according to the manufacturer's instructions. Heart rate was recorded continuously throughout using short-range telemetry (Polar S810, Finland). Breath-by-breath data were averaged every 30-seconds and $VO_{2\ peak}$, the objective measure of cardiorespiratory fitness, was recorded as the average oxygen uptake across the final 15-seconds before the termination of the test. In addition, self-reported physical activity levels were recorded using the International Physical Activity Questionnaire [IPAQ](Craig *et al.*, 2003).

For Review Only

Statistical analyses

An initial quality assessment removed any statistical outliers, defined as more than 3 standard deviations from the mean. To avoid biasing the results, all participants were included in the statistical analysis, including when missing data was present. A linear mixed effects model was used to assess change in cardiorespiratory physiology, cognition, and cerebral perfusion in *a priori* defined region of interests (ROIs) in R (version 1.1.463)(R Core Team, 2016) using the lme4 package(Bates *et al.*, 2015); between-subject effect: genotype [controls, HD], within-subject effect: time [change from baseline at timepoints post1, post2, post3] and hemisphere for perfusion analysis [left and right]; p-values were calculated from degrees of freedom estimated using Satterthwaite's method(Kuznetsova *et al.*, 2016). One advantage of a mixed effect model is the ability to estimate fixed effects from incomplete data(West and Galecki, 2012). For the perfusion analysis, $PETCO_2$ and age were demeaned and included as covariates in the model along with sex. Post-hoc analyses examined HD and control participants separately to assess the effect of exercise.

Where a significant genotype effect on CBF was found, a follow up regression model was built from a set of a priori candidate predictor variables by entering and removing predictors based on Akaike Information Criteria, in a stepwise manner. The a priori predictors entered were: a genetic predictor (CAG repeat length), a clinical predictor (UHDRS TMS), a performance-related predictor (average heart rate reserve [HRR] achieved during the intervention), and a physiological predictor ($PETCO_2$ change, known to modulate CBF).

For the cognitive tests with a motor component (Trail Making, SCOLP, Symbol Digit Modalities Test), motor speed was included as a covariate in the analyses. Age was a covariate in all cognitive analyses.

To compare performance and the physiological and perceptual response during the exercise intervention, heart rate, lactate, ratings of perceived exertion, work rate and cycling speed were

For Review Only

averaged across the intervention period, excluding the warm up and warm down period, and compared between HD and control participants. All data are expressed as mean ± SEM unless stated and are available on request.

Data availability

The CBF imaging data, physiological data, data analysis scripts and R code used for statistical analysis will be made available upon request for academic and non-commercial purposes.

For Review Only

Results

HD and control participants did not differ on gender, age, body mass index (BMI), fitness levels (measured using a VO_2 peak test) and self-reported physical activity levels (all $p > 0.05$, Table 1). Unadjusted values are shown in Supplementary Table 1. The cerebrovascular reactivity data was not analysed due to insufficient statistical power (see Supplementary Information).

Physiological and self-reported response to the exercise intervention

During the exercise intervention, heart rate (HR) was significantly elevated compared to the upright rest period on the ergometer for control and HD participants (exercise effect, $p < 0.001$), with control participants working at an average 54.8 ± 6.1 % of their maximum, calculated using heart rate reserve, compared to 54.8 ± 10.3 % for HD participants (genotype effect, $p = 0.99$). Average heart rate did not differ across the intervention for controls (126.8 ± 2.8 beats/min) and HD participants (127.2 ± 3.3 beats/min).

Mean lactate concentration across the 20-minute intervention did not significantly differ between control participants (2.87 ± 0.22 mmoL) and HD participants (2.23 ± 0.27 mmoL; p

= 0.07). Mean self-reported ratings of exertion were associated with the verbal anchor 'moderate' to 'somewhat hard' exertion on the Borg 0-10 RPE scale for both controls and HD participants in the legs (controls = 3.89 ± 0.22; HD = 4.25 ± 0.50, $p = 0.53$) and for breathing (controls = 3.38 ± 0.21; HD = 3.44 ± 0.42, $p = 0.89$). Results across the time course of the intervention are shown in Supplementary Figure 1.

Despite no differences in the physiological or self-reported response to the intervention, HD participants cycled at a significantly lower workload (60.93 ± 5.09 watts) compared to controls (90.86 ± 4.77 watts, $F_{1,35} = 18.29$, $p < 0.001$, $\eta^2 = 0.34$), whereas there was no group difference in cycling speed (controls = 69.67 ± 2.45 revolutions/min; HD participants = 71.63 ± 3.03 revolutions/min, $p = 0.62$).

Mean heart rate, MAP and $PETCO_2$ values for the baseline and post-exercise scan session are shown in Table 2. Exercise and genotype did not interact on any measure (all $p > 0.05$). Exercise and genotype had a significant main effect on heart rate; HD participants' heart rate was 7.77 ± 3.61 beats/min higher compared to controls (p=0.037). After the exercise session, heart rate remained elevated across both groups at 15-minutes (+7.88 ± 1.23 beats/min, p = 5.23e-09), and 40-minutes post exercise (+2.50 ± 1.18 beats/min, $p = 0.037$), before returning to baseline levels after 60-minutes (+1.46 ± 1.21 beats/min, $p = 0.22$).

Exercise and genotype had no effect on MAP, with no evidence of exercise-induced hypotension when the post-exercise scan session began ($p > 0.05$).

$PETCO_2$ levels were similar for HD and control participants, and exercise-induced hypocapnia was observed across both groups of participants and persisted for 60-minutes post exercise, with a significant reduction in $PETCO_2$ from baseline at 15-minutes ($t_{88.5} = -2.48$, $p = 0.015$), 40-minutes ($t_{88.5} = -2.96$, $p = 0.004$) and 60-minutes post exercise ($t_{88.3} = -2.19$, $p = 0.03$), see Figure 2H.

For Review Only

Regionally-specific effects of exercise on blood flow in HD

Absolute CBF values (estimated marginal means adjusted for age, sex and end-tidal CO_2) are reported in Table 2; the change in cerebral blood flow (CBF) from baseline is shown in Figure 2 for the three post exercise time points (15 minutes, 40 minutes, 60 minutes).

Globally, exercise (p=0.80) and genotype (p=0.73) had no effect on grey matter CBF. A genotype *x* exercise interaction was found in the middle frontal gyrus and precentral gyrus; forty minutes after exercise cessation the exercise-induced CBF change from baseline was 13.67 ± 6.75 ml/100g/min higher in HD patients in the middle frontal gyrus ($t_{81.8} = 2.03$, p = 0.046, Figure 2B) and 14.12 ± 5.71 ml/100g/min higher in the precentral gyrus in HD participants compared to controls ($t_{81.1} = 2.47$, p = 0.016, Figure 2C). Post-hoc analyses found the change in CBF relative to baseline in the precentral and middle frontal gyrus was significant in the HD participants (precentral increase = 11.73 ± 4.36 ml/100g/min, p = 0.010, MFG increase = 14.88 ± 5.65 ml/100g/min, p = 0.012), whereas the change was not significant in control participants (precentral decrease = -4.61 ± 4.33, p =0.29, MFG decrease= -2.25 ± 4.53, p =0.62).

Controls and HD participants did not differ in precentral and middle frontal gyrus CBF at baseline (p=0.60 and 0.32 respectively), 15-minutes post exercise (6.20 ± 5.52 and 2.60 ± 6.54 ml/100g/min respectively) and 60-minutes post exercise (6.77 ± 5.96, and 0.89 ± 7.03 ml/100g/min; all p > 0.05). Exercise and genotype had no effect on CBF values in the postcentral gyrus.

In the subcortical ROIs, hippocampal CBF was 5.12 ± 2.45 ml/100g/min higher 60-minutes post exercise across all participants ($t_{205} = 2.07$, p = 0.039). Additionally, an interaction between exercise and genotype was found ($F_{3,198} = 3.05$, p = 0.03); at 40-minutes post exercise, the exercise-induced CBF change was significantly different between HD and control participants

For Review Only

(6.98 ± 3.51 ml/100g/min; t_{200} = 1.99, p = 0.048, see Figure 2G). Post-hoc analysis found this was driven by a significant increase in hippocampal CBF in HD participants 40-minutes after exercise cessation (+6.53 ± 3.15 ml/100g/min, p = 0.04), whereas there was no significant change in control participants at this time (1.28 ± 2.30, p = 0.58). Baseline hippocampal CBF did not differ between controls and HD participants (t_{68} = 0.12, p = 0.90).

Exercise and genotype had no effect on CBF in the thalamus or caudate, and no effect on arterial arrival time (AAT) averaged across the grey matter or in any of the ROIs (all p > 0.05, see Supplementary Table 2).

For Review Only

Predictors of the exercise-induced CBF response

VO_2 peak was not correlated with baseline, resting cerebral blood flow in grey matter or in the specified ROIs and genotype did not affect the relationship (all p> 0.05).

A regression model was used to examine the predictors of the observed perfusion changes seen 40-minutes after exercise cessation; the scaled estimates for each predictor in the regression model for HD and control participants are shown in Figure 3.

In HD participants, HRR, $PETCO_2$ change and TMS were entered into the regression model and explained 68.2% of the variance in exercise-induced CBF change in the hippocampus (p = 0.0001), with HRR explaining 42.8% of the variance alone (β = -148.8, p <0.001), whilst $PETCO_2$ and TMS were non-significant predictors (β = -0.62 and -0.35, p = 0.129 and 0.086 respectively, see Figure 3).

In the precentral gyrus, 57% of the variance in CBF change was explained by CAG repeat length (p = 0.031, β = -4.17), $PETCO_2$ change (p = 0.022, β = 2.38) and HRR during the intervention (p=0.97, β = -1.62) whilst the clinical predictor (TMS) did not meet the criteria for entry into the regression model.

In the MFG, $PETCO_2$ change (β = 3.20, p = 0.06), CAG repeat length change (β = -4.26, p =0.16), and HRR change (β = 17.26, p = 0.80), were entered into the model which combined explained 29.1% of the CBF variance and was not significant (p=0.18).

In control participants at the same timepoint 40-minutes post exercise cessation, HRR and $PETCO_2$ were not predictive of hippocampal CBF change (adjusted R^2 = 0.05, p = 0.20), MFG CBF change (adjusted R^2 = -0.13, p = 0.76) or precentral CBF change (adjusted R^2 = -0.08, p = 0.62).

Cognitive performance is impaired following acute exercise

Cognitive test performance is shown in **Error! Reference source not found.**3; HD participants performed worse on all cognitive tests (main effect of gene status, all $p < 0.05$) and had a significantly slower motor tapping speed compared to controls ($F_{1,33}$ = 17.06, p = 0.0002).

A main effect of exercise was found for performance on the Stroop interference task (p = 0.002), symbol digits modality test (p = 0.007) and speed of comprehension SCOLP test (p=0.003), with worse performance observed following the exercise intervention compared to baseline (see Table 3). There was no interaction between the effect of exercise and gene status.

For Review Only

Discussion

Long-term exercise interventions improve motor functioning in people with HD(Khalil *et al.*, 2013; Quinn *et al.*, 2016; Fritz *et al.*, 2017) although the underlying mechanism is poorly understood. Here, using a highly controlled acute exercise paradigm, we aimed to characterise the cerebrovascular response to a single session of aerobic exercise in people with HD using arterial spin labelling (ASL)-MRI. Our data showed that twenty-minutes of moderate intensity cycling induced a transient regionally-selective cerebral perfusion response that was different in HD participants compared to control participants. Whilst exercise induced a non-significant

negative change in cerebral blood flow (CBF) in control participants in the precentral and middle frontal gyri, a significant increase in CBF was observed in HD participants 40-minutes after exercise cessation. Likewise, in the hippocampus, an area previously shown to be selectively responsive to acute exercise(Steventon *et al.*, 2019), CBF was significantly elevated 40-minutes after exercise in HD participants compared to controls. We further observed an increase in hippocampal blood flow 60-minutes after exercise cessation in both controls and HD participants, in line with previous work using a similar methodological approach(Steventon *et al.*, 2019).

The differential effect of exercise on cerebral perfusion in HD participants compared to controls was hypothesised based on an altered cardiovascular response to exercise(Steventon *et al.*, 2018) and resting cerebrovascular abnormalities(Drouin-Ouellet *et al.*, 2015), however the direction of perfusion effects in HD participants was not anticipated, with an increase in CBF generally interpreted as a beneficial effect in healthy cohorts. However, given that an increase in CBF was not also seen in controls, the cerebrovascular response more likely reflects a latent pathology induced by exercise in HD participants. In support of this, cerebral hyper-perfusion has been documented previously in HD patients and animal models, observed as increased vessel density, increased cerebral blood volume and flow, increased blood brain barrier permeability and greater release of VEGF-A, an angiogenic growth factor, by astrocytes(Vis *et al.*, 1998; Harris *et al.*, 1999; Wolf *et al.*, 2011; Chen *et al.*, 2012; Franciosi *et al.*, 2012; Lin *et al.*, 2013; Hua *et al.*, 2014; Hsiao *et al.*, 2015). Increased angiogenesis coupled with a reduced number of pericytes and altered vascular reactivity has been observed in HD mice(Hsiao *et al.*, 2015), suggesting a complex functional impairment which may impact neurovascular coupling and thus the cerebrovascular response to exercise. Genetic load (CAG repeat length) was predictive of the cerebrovascular response to exercise in HD participants in the precentral gyrus, with a larger post-exercise increase in CBF observed in participants with

For Review Only

a lower CAG repeat length. Nevertheless, further research is warranted to test whether the increase in CBF could alternatively reflect a compensatory mechanism to support neuronal survival, as it may be that certain regions are less vulnerable to hindered cerebral haemodynamics.

The regional specificity of the exercise effects are particularly noteworthy and are partly in agreement with previous work in young adults and stroke patients(MacIntosh *et al.*, 2014; Robertson *et al.*, 2015; Steventon *et al.*, 2019), suggesting the observed effects are specific to the exercise intervention in the HD group. The precentral gyrus, also known as the primary motor cortex, is a vital structure involved in executing voluntary motor movements and a substantial body of literature shows the motor system is capable of cortical functional reorganisation(Wall *et al.*, 2002; Lee *et al.*, 2003) and acute compensatory plasticity, with changes in functional organisation demonstrated over minutes, weeks, and longer durations(Wall *et al.*, 2002; Lee *et al.*, 2003; Weiss *et al.*, 2004; Björkman *et al.*, 2009). The regional effects were not explained by baseline resting hypo- or hyper-perfusion in the HD participants, however previous work has shown significant cortical thinning and locally decreased task related fMRI activation in both the middle frontal and precentral gyri in HD participants(Wolf *et al.*, 2007, 2008; Rosas *et al.*, 2008; Saft *et al.*, 2008), which further support the concept that the regionally-selective effect in HD participants and not controls are due to underlying vascular alterations in HD, which convey an increased propensity for exercise-induced vascular adaptions.

The regulation of CBF is controlled by neurogenic, metabolic, autoregulatory and systemic factors and exercise-induced changes in CBF may be accounted for by changes in some or all of these in HD participants. However, the temporal pattern of cardiorespiratory recovery after exercise cannot explain our results; heart rate, blood pressure and the end-tidal partial pressure of carbon dioxide ($PETCO_2$) were not differentially affected by exercise in HD

For Review Only

and control participants. In both groups, blood pressure recovered to baseline levels prior to the post exercise scans and heart rate was elevated during both the 15- and 40-minutes post exercise scan, whereas a CBF difference was only observed at 40-minutes. $PETCO_2$ remained significantly lower than baseline at all three post-exercise timepoint, and because $PETCO_2$ is known to modulate arteriolar diameter(Thomas *et al.*, 1989; Ide and Secher, 2000; Ratanakorn *et al.*, 2001) it was accounted for in the statistical model, and was found to predict the CBF change in the precentral gyrus in HD participants. However, this is unlikely to explain the differences seen between controls and HD participants as the magnitude of $PETCO_2$ change did not differ between the two groups and $PETCO_2$ did not account for the changes observed in the hippocampus and middle frontal gyrus. In a healthy vascular system, hypocapnia (such as that observed here following exercise) causes an increase in cerebral vasoconstriction, therefore a reduction in CBF would be expected, rather than the observed increase in CBF seen in HD participants. However, impaired vascular reactivity to haemodynamic challenges has been shown in HD mouse models, with a smaller increase in CBF following carbogen in HD mice compared to wild types, despite greater vessel density(Hsiao *et al.*, 2015). Thus, the observed increase in CBF after exercise in HD patients may be due to a blunted hypocapnic response in HD participants, although the results from the regression model do not support this in all of the regions affected. We intended to measure the CBF response to hypercapnia in this study to test this hypothesis, however the breath-hold paradigm we utilised was limited by highly variable and poor task performance in both groups and thus requires further optimisation before a robust measure of cerebrovascular reactivity can be obtained.

The temporal specificity of our results is particularly novel, with a difference between HD and controls observed 40-minutes after exercise cessation, but not at 15- and 60-minutes post exercise. One explanation may that the CBF difference seen at 40-minutes between HD and control participants is due to a lag effect from the CVR breath-hold challenge, performed

For Review Only

immediately before the 40-minute CBF scan, as during breath-holding, the increase in the partial pressure of CO_2 gives rise to increased CBF because of vasomotor reactivity. However, this is unlikely due to the poor performance of both groups of participants during the breath hold challenge, with CO_2 increasing after a breath-hold on only half of the trials. More likely, this result suggests there is a temporal window for acute exercise effects which may be driven by resting vascular perturbations in the HD participants. In support of this, work in stroke patients show a transient time-dependent CBF response to exercise in distinct brain regions, with a reduction in CBF in the middle frontal gyrus observed 30-minutes post exercise, and returning to baseline by 50-minutes(Robertson *et al.*, 2015).

Designing an exercise intervention that was physically and perceptually similar for the patient and control group was challenging given that HD patients have an altered metabolic and cardiorespiratory response to submaximal exercise(Steventon *et al.*, 2018). Nevertheless, we achieved an equivalent moderate-intensity aerobic intervention with no difference in the peripheral physiological response or self-reported exertion, suggesting that the observed effects in HD are not due to a difference in the intervention prescription. However, HD participants on average cycled at a lower workload in order to achieve the prescribed target heart rate, most likely due to an altered movement economy. The exercise intensity achieved by HD participants during the intervention was found to predict the hippocampal perfusion response, which may indicate that unlike the cortical regions, the exercise-induced hippocampal effect is intensity-dependent.

Functional improvements in cognition have been reported following long-term exercise interventions in healthy cohorts(Dustman *et al.*, 1984; Kramer *et al.*, 1999) as well as in Parkinson's disease(Duchesne *et al.*, 2015; Altmann *et al.*, 2016) and Alzheimer's disease(Ströhle *et al.*, 2015; Teixeira *et al.*, 2018). In this study, cognitive performance approximately one-hour after exercise cessation was worse than at baseline in HD and control

For Review Only

participants alike, most likely due to fatigue associated with the lengthy testing session, rather than a direct effect of exercise. Whereas baseline cognitive tests were completed at the beginning of the experimental session on a separate day, the post-exercise testing was conducted approximately 3.5 hours into the experimental session. Cognitive data were a secondary outcome measure and it is plausible that transient cognitive gains returned to baseline levels by the time of testing, with the study not optimally designed to examine transient cognitive effects and appropriately control for fatigue. We were also likely underpowered to detect an effect, with acute gains in cognition previously reported with small effect sizes(Chang *et al.*, 2012*b*).

Conclusions

Overall, we observed a differential response to a single session of exercise in HD and control participants, with a transient regionally-selective increase in perfusion in HD participants. The highly controlled acute exercise paradigm used may provide a framework for determining the key components that enable exercise to modulate a pathologically disturbed cerebrovasculature for therapeutic gain in HD. Further work is necessary to understand the extent and pattern of disruptions to the neurovascular unit and blood brain barrier in HD to inform the development of targeted exercise approaches.

Acknowledgments

Background data was used from Enroll-HD, a clinical research platform and longitudinal observational study for Huntington's disease families intended to accelerate progress towards therapeutics; it is sponsored by CHDI Foundation, a nonprofit biomedical research organization exclusively dedicated to collaboratively developing therapeutics for HD. Enroll-

For Review Only

HD would not be possible without the vital contribution of the research participants and their families. We also wish to acknowledge the individuals who contributed to the collection of the Enroll-HD data (https://www.enroll-hd.org/acknowledgments/).

Funding

JS and KM were supported by the Wellcome Trust (200804/Z/16/Z). Data was used from the Enroll-HD database, which is a clinical research platform and longitudinal observational study sponsored by CHDI Foundation.

Competing Interests

The authors report no competing interests in relation to the work described.

For Review Only

Supplementary Data

Cerebrovascular reactivity breath-hold challenge

During the CVR breath-hold challenge, participants were instructed to complete 10 breath-holds. A successful breath-hold performance was defined as: (a) a normalised $PETCO_2$ peak from Fourier transform over the $PETCO_2$ time course greater than or equal to 10, and (b) increased $PETCO_2$ on exhalation following the breath-hold on more than half of the trials. Analysis of the end-tidal CO2 traces show that performance was poor in both HD participants (5.4 ± 0.8 successful breath holds) and control participants (4.8 ± 0.8 successful breath holds); 11 HD participants and 15 control participants did not meet the criteria for a successful breath-hold challenge, whilst two controls and three HD participants did not complete the challenge at both timepoints.

SUPPLEMENTARY TABLE 1 UNADJUSTED VALUES FOR CARDIORESPIRATORY AND CEREBRAL MEASURES, STRATIFIED FOR GENOTYPE AND TIME RELATIVE TO THE EXERCISE INTERVENTION. HR: HEART RATE. MAP: MEAN ARTERIAL PRESSURE, MFG: MIDDLE FRONTAL GYRUS

	Baseline				Post 1				Post 2				Post 3			
	Controls		HD		Controls		HD		Controls		HD		Controls		HD	
	Mean	SD	Mean	SD	Mean	SD	Mean	SD	Mean	SD	Mean	SD	Mean	SD	Mean	SD
HR, beats/min	62.0	9.8	69.8	11.3	69.9	10.2	77.8	11.4	65.2	9.2	72.2	9.4	64.5	10.2	74.2	10.9
$PETCO_2$, mmHg	36.3	5.1	34.9	5.6	34.4	4.8	31.6	5.3	34.0	4.9	31.3	5.4	34.6	4.4	32.0	5.3
MAP, mmHg	88.1	12.1	92.7	9.0	86.9	12.3	90.2	7.8	87.2	14.2	89.9	11.2	87.9	12.8	92.5	9.7
CBF (ml/100g/min)																
GM	53.2	15.0	49.6	11.0	53.3	11.5	52.0	12.8	50.9	17.2	52.1	11.0	53.2	12.8	49.2	11.3
Precentral Gyrus	55.3	17.0	49.5	16.1	52.2	13.7	52.4	16.6	50.4	17.5	55.9	17.6	52.2	17.3	49.8	16.8
Postcentral Gyrus	52.3	17.8	45.4	18.6	52.0	14.2	49.7	16.8	51.4	21.1	53.2	18.7	56.1	25.6	46.7	17.1
MFG	44.9	15.6	36.4	18.0	42.4	11.9	37.8	16.1	42.3	21.0	43.3	21.1	42.9	16.8	34.7	17.0
Hippocampus	48.7	13.8	48.7	12.9	49.9	12.8	51.0	17.2	48.6	15.5	54.6	13.2	54.2	9.8	47.9	12.0
Thalamus	45.3	13.4	40.8	13.7	45.6	16.7	42.9	17.4	42.7	19.0	45.9	18.8	41.7	11.7	40.7	16.6
Caudate	30.5	12.6	24.3	9.0	29.2	9.4	26.0	16.2	29.6	11.9	26.0	9.6	29.4	13.1	25.0	12.3
**Arterial arrival time (seconds)**																
GM	0.75	0.03	0.74	0.03	0.74	0.04	0.74	0.04	0.74	0.04	0.72	0.04	0.74	0.06	0.74	0.04
Precentral	0.87	0.08	0.84	0.09	0.86	0.09	0.85	0.10	0.85	0.09	0.80	0.10	0.83	0.11	0.83	0.08
Postcentral	0.85	0.09	0.84	0.09	0.85	0.09	0.85	0.10	0.85	0.09	0.79	0.09	0.82	0.11	0.81	0.08
MFG	0.91	0.11	0.84	0.13	0.88	0.10	0.89	0.13	0.89	0.10	0.84	0.14	0.88	0.14	0.83	0.10
Hippocampus	0.66	0.04	0.67	0.04	0.66	0.04	0.66	0.04	0.65	0.05	0.65	0.05	0.66	0.04	0.67	0.04
halamus	0.79	0.07	0.77	0.07	0.77	0.07	0.78	0.07	0.76	0.08	0.75	0.09	0.76	0.10	0.79	0.07
Caudate	0.72	0.05	0.71	0.06	0.73	0.08	0.73	0.07	0.73	0.06	0.72	0.09	0.71	0.06	0.73	0.06

For Review Only

Supplementary Table 2. Estimated arterial arrival time (AAT, seconds) in grey matter and ROIs. Data shown are mean ± standard error of the mean. CON: controls.

	Baseline		Post exercise scan session					
			Post 1		Post 2		Post 3	
	CON	HD	CON	HD	CON	HD	CON	HD
AAT (s) in ROIs								
Grey matter	0.75 ± 0.008	0.74 ± 0.008	0.74 ± 0.009	0.74 ± 0.010	0.74 ± 0.009	0.73 ± 0.010	0.74 ± 0.012	0.74 ± 0.012
Middle frontal gyrus	0.91 ± 0.027	0.84 ± 0.029	0.88 ± 0.027	0.89 ± 0.029	0.89 ± 0.027	0.85 ± 0.029	0.88 ± 0.031	0.83 ± 0.032
Postcentral gyrus	0.85 ± 0.020	0.84 ± 0.022	0.85 ± 0.022	0.85 ± 0.024	0.85 ± 0.020	0.80 ± 0.021	0.82 ± 0.024	0.81 ± 0.025
Precentral gyrus	0.87 ± 0.020	0.84 ± 0.021	0.86 ± 0.021	0.85 ± 0.023	0.85 ± 0.021	0.81 ± 0.022	0.83 ± 0.025	0.83 ± 0.026
Caudate	0.72 ± 0.010	0.71 ± 0.011	0.73 ± 0.015	0.72 ± 0.016	0.73 ± 0.015	0.74 ± 0.016	0.71 ± 0.013	0.73 ± 0.013
Thalamus	0.79 ± 0.015	0.77 ± 0.017	0.77 ± 0.015	0.78 ± 0.017	0.76 ± 0.019	0.76 ± 0.020	0.76 ± 0.021	0.78 ± 0.022
Hippocampus	0.66 ± 0.009	0.68 ± 0.010	0.66 ± 0.008	0.66 ± 0.009	0.65 ± 0.010	0.65 ± 0.010	0.66 ± 0.008	0.67 ± 0.008

For Review Only

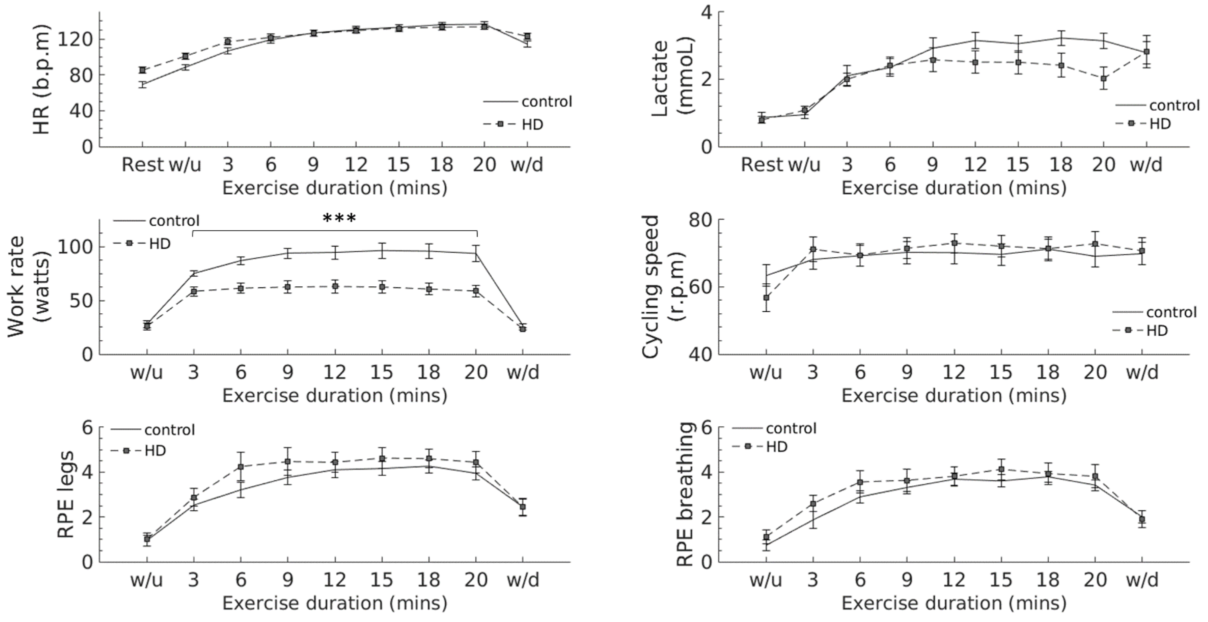

Supplementary Figure 1. Physiological, perceptual and performance response to the 20-minute exercise intervention. w/u : warm-up period, w/d: warm-down period. HR: heart rate; RPE: rating of perceived exertion [10-point scale]. Data are means ± S.E.M. *** $p < 0.001$.

For Review Only

References

Altmann LJP, Stegemöller E, Hazamy AA, Wilson JP, Bowers D, Okun MS, et al. Aerobic Exercise Improves Mood, Cognition, and Language Function in Parkinson's Disease: Results of a Controlled Study. J Int Neuropsychol Soc 2016; 22: 878–889.

American College of Sports Medicine., Tharrett SJ, Peterson JA. ACSM's health/fitness facility standards and guidelines. Human Kinetics; 2012

Bates D, Maechler M, Bolke B, Walker S. Fitting linear mixed-effects models using lme4. J. Stat. Softw. 67, 1 – 48. (doi:10. 18637/jss.v067.i01). J Stat Softw 2015; 67: 1 – 48.

Bates G, Harper PS, Jones L. Huntington's disease. 3rd ed. New York: Oxford University Press; 2002

Björkman A, Weibull A, Rosén B, Svensson J, Lundborg G. Rapid cortical reorganisation and improved sensitivity of the hand following cutaneous anaesthesia of the forearm. Eur J Neurosci 2009; 29: 837–44.

Borg GA. Perceived exertion. Exerc Sport Sci Rev 1974; 2: 131–53.

Chang YK, Labban JD, Gapin JI, Etnier JL. The effects of acute exercise on cognitive performance: A meta-analysis. Brain Res 2012; 1453: 87–101.

Chang YK, Labban JD, Gapin JI, Etnier JL. The effects of acute exercise on cognitive performance: A meta-analysis. Brain Res 2012; 1453: 87–101.

Chappell MA, Groves AR, MacIntosh BJ, Donahue MJ, Jezzard P, Woolrich MW. Partial volume correction of multiple inversion time arterial spin labeling MRI data. Magn Reson Med 2011; 65: 1173–83.

Chappell MA, MacIntosh BJ, Donahue MJ, Günther M, Jezzard P, Woolrich MW. Separation

For Review Only

of macrovascular signal in multi-inversion time arterial spin labelling MRI. Magn Reson Med 2010; 63: 1357–1365.

Chen JJ, Salat DH, Rosas HD. Complex relationships between cerebral blood flow and brain atrophy in early Huntington's disease. Neuroimage 2012; 59: 1043–1051.

Coles K, Tomporowski PD. Effects of acute exercise on executive processing, short-term and long-term memory. J Sports Sci 2008; 26: 333–44.

Cox RW. AFNI: software for analysis and visualization of functional magnetic resonance neuroimages. Comput Biomed Res 1996; 29: 162–73.

Craig CL, Marshall AL, Sjöström M, Bauman AE, Booth ML, Ainsworth BE, et al. International physical activity questionnaire: 12-country reliability and validity. Med Sci Sports Exerc 2003; 35: 1381–95.

Cruickshank TM, Thompson JA, Domínguez D JF, Reyes AP, Bynevelt M, Georgiou-Karistianis N, et al. The effect of multidisciplinary rehabilitation on brain structure and cognition in Huntington's disease: an exploratory study. Brain Behav 2015; 5: n/a-n/a.

van Dellen A, Blakemore C, Deacon R, York D, Hannan AJ. Delaying the onset of Huntington's in mice. Nature 2000; 404: 721–2.

van Dellen A, Cordery PM, Spires TL, Blakemore C, Hannan AJ. Wheel running from a juvenile age delays onset of specific motor deficits but does not alter protein aggregate density in a mouse model of Huntington's disease. BMC Neurosci 2008; 9: 34.

Drouin-Ouellet J, Sawiak SJ, Cisbani G, Lagacé M, Kuan W-L, Saint-Pierre M, et al. Cerebrovascular and blood-brain barrier impairments in Huntington's disease: Potential implications for its pathophysiology. Ann Neurol 2015; 78: 160–177.

Duchesne C, Lungu O, Nadeau A, Robillard ME, Boré A, Bobeuf F, et al. Enhancing both

For Review Only

motor and cognitive functioning in Parkinson's disease: Aerobic exercise as a rehabilitative intervention. Brain Cogn 2015; 99: 68–77.

Dustman RE, Ruhling RO, Russell EM, Shearer DE, Bonekat HW, Shigeoka JW, et al. Aerobic exercise training and improved neuropsychological function of older individuals. Neurobiol Aging 1984; 5: 35–42.

Franciosi S, Ryu JK, Shim Y, Hill A, Connolly C, Hayden MR, et al. Age-dependent neurovascular abnormalities and altered microglial morphology in the YAC128 mouse model of Huntington disease. Neurobiol Dis 2012; 45: 438–49.

Fritz NE, Rao AK, Kegelmeyer D, Kloos A, Busse M, Hartel L, et al. Physical Therapy and Exercise Interventions in Huntington's Disease: A Mixed Methods Systematic Review. J Huntingtons Dis 2017; 6: 217–235.

Gusella JF, MacDonald ME, Ambrose CM, Duyao MP. Molecular genetics of Huntington's disease. Arch Neurol 1993; 50: 1157–63.

Harris GJ, Codori AM, Lewis RF, Schmidt E, Bedi A, Brandt J. Reduced basal ganglia blood flow and volume in pre-symptomatic, gene-tested persons at-risk for Huntington's disease. Brain 1999; 122 (Pt 9): 1667–78.

Harrison DJ, Busse M, Openshaw R, Rosser AE, Dunnett SB, Brooks S. Exercise attenuates neuropathology and has greater benefit on cognitive than motor deficits in the R6/1 Huntington's disease mouse model. Exp Neurol 2013; 248: 457–469.

Herbst EAF, Holloway GP. Exercise training normalizes mitochondrial respiratory capacity within the striatum of the R6/1 model of Huntington's disease. Neuroscience 2015; 303: 515–23.

Hsiao H-Y, Chen Y-C, Huang C-H, Chen C-C, Hsu Y-H, Chen H-M, et al. Aberrant astrocytes impair vascular reactivity in Huntington disease. Ann Neurol 2015; 78: 178–192.

For Review Only

Hua J, Unschuld PG, Margolis RL, van Zijl PCM, Ross CA. Elevated arteriolar cerebral blood volume in prodromal Huntington's disease. Mov Disord 2014; 29: 396–401.

Ide K, Secher NH. Cerebral blood flow and metabolism during exercise. Prog Neurobiol 2000; 61: 397–414.

Jenkinson M, Beckmann CF, Behrens TEJ, Woolrich MW, Smith SM. FSL. Neuroimage 2012; 62: 782–90.

Jo JS, Chen J, Riechman S, Roig M, Wright DL. The protective effects of acute cardiovascular exercise on the interference of procedural memory [Internet]. Psychol Res 2018[cited 2018 Dec 5] Available from: http://www.ncbi.nlm.nih.gov/pubmed/29637259

Khalil H, Quinn L, van Deursen R, Dawes H, Playle R, Rosser A, et al. What effect does a structured home-based exercise programme have on people with Huntington's disease? A randomized, controlled pilot study. Clin Rehabil 2013; 27: 646–58.

Kilroy E, Apostolova L, Liu C, Yan L, Ringman J, Wang DJJ. Reliability of two-dimensional and three-dimensional pseudo-continuous arterial spin labeling perfusion MRI in elderly populations: Comparison with 15o-water positron emission tomography. J Magn Reson Imaging 2014; 39: 931–939.

Kramer AF, Hahn S, Cohen NJ, Banich MT, McAuley E, Harrison CR, et al. Ageing, fitness and neurocognitive function. Nature 1999; 400: 418–419.

Kuznetsova A, Brockhof P, Christensen R. lmerTest: Tests in linear mixed effects models. 2016

Lee L, Siebner HR, Rowe JB, Rizzo V, Rothwell JC, Frackowiak RSJ, et al. Acute remapping within the motor system induced by low-frequency repetitive transcranial magnetic stimulation. J Neurosci 2003; 23: 5308–18.

For Review Only

Lezak MD. Neuropsychological assessment [Internet]. Oxford University Press; 2012[cited 2017 Sep 20] Available from: https://global.oup.com/academic/product/neuropsychological-assessment-9780195395525?cc=gb&lang=en&

Lin C-Y, Hsu Y-H, Lin M-H, Yang T-H, Chen H-M, Chen Y-C, et al. Neurovascular abnormalities in humans and mice with Huntington's disease. Exp Neurol 2013; 250: 20–30.

MacIntosh BJ, Crane DE, Sage MD, Rajab AS, Donahue MJ, McIlroy WE, et al. Impact of a single bout of aerobic exercise on regional brain perfusion and activation responses in healthy young adults. PLoS One 2014; 9: e85163.

Mang CS, Snow NJ, Campbell KL, Ross CJD, Boyd LA. A single bout of high-intensity aerobic exercise facilitates response to paired associative stimulation and promotes sequence-specific implicit motor learning. J Appl Physiol 2014; 117: 1325–1336.

Pang TYC, Stam NC, Nithianantharajah J, Howard ML, Hannan AJ. Differential effects of voluntary physical exercise on behavioral and brain-derived neurotrophic factor expression deficits in huntington's disease transgenic mice. Neuroscience 2006; 141: 569–584.

Paulsen JS, Nopoulos PC, Aylward E, Ross CA, Johnson H, Magnotta VA, et al. Striatal and white matter predictors of estimated diagnosis for Huntington disease. Brain Res Bull 2010; 82: 201–7.

Pereira AC, Huddleston DE, Brickman AM, Sosunov AA, Hen R, McKhann GM, et al. An in vivo correlate of exercise-induced neurogenesis in the adult dentate gyrus. Proc Natl Acad Sci U S A 2007; 104: 5638–43.

van Praag H, Shubert T, Zhao C, Gage FH. Exercise enhances learning and hippocampal neurogenesis in aged mice. J Neurosci 2005; 25: 8680–5.

Quinn L, Hamana K, Kelson M, Dawes H, Collett J, Townson J, et al. A randomized, controlled trial of a multi-modal exercise intervention in Huntington's disease. Parkinsonism

For Review Only

Relat Disord 2016; 31: 46–52.

R Core Team T. R: a language and environment for statistical computing. 2016

Rahman A, Ekman M, Shakirova Y, Andersson KE, Mörgelin M, Erjefält JS, et al. Late onset vascular dysfunction in the R6/1 model of Huntington's disease. Eur J Pharmacol 2013; 698: 345–353.

Ratanakorn D, Greenberg JP, Meads DB, Tegeler CH. Middle cerebral artery flow velocity correlates with common carotid artery volume flow rate after CO2 inhalation. J Neuroimaging 2001; 11: 401–5.

Reitan RM. Manual for administration and scoring of the Halstead–Reitan of neuropsychological test battery for adults and children. Tucson: Neuropsychology Press.; 1979

Robertson AD, Crane DE, Rajab AS, Swardfager W, Marzolini S, Shirzadi Z, et al. Exercise intensity modulates the change in cerebral blood flow following aerobic exercise in chronic stroke. Exp Brain Res 2015; 233: 2467–2475.

Roig M, Skriver K, Lundbye-Jensen J, Kiens B, Nielsen JB. A Single Bout of Exercise Improves Motor Memory. PLoS One 2012; 7: e44594.

Rosas HD, Salat DH, Lee SY, Zaleta AK, Pappu V, Fischl B, et al. Cerebral cortex and the clinical expression of Huntington's disease: complexity and heterogeneity. Brain 2008; 131: 1057–68.

Saft C, Schüttke A, Beste C, Andrich J, Heindel W, Pfleiderer B. fMRI reveals altered auditory processing in manifest and premanifest Huntington's disease. Neuropsychologia 2008; 46: 1279–89.

Shoulson I, Fahn S. Huntington disease: clinical care and evaluation. Neurology 1979; 29: 1–

For Review Only

3.

Skriver K, Roig M, Lundbye-Jensen J, Pingel J, Helge JW, Kiens B, et al. Acute exercise improves motor memory: exploring potential biomarkers. Neurobiol Learn Mem 2014; 116: 46–58.

Smith JC, Paulson ES, Cook DB, Verber MD, Tian Q. Detecting changes in human cerebral blood flow after acute exercise using arterial spin labeling: Implications for fMRI. J Neurosci Methods 2010; 191: 258–262.

St-Amour I, Aubé B, Rieux M, Cicchetti F. Targeting cerebrovascular impairments in Huntington's disease: a novel treatment perspective. Neurodegener Dis Manag 2015; 5: 389–393.

Steventon JJ, Collett J, Furby H, Hamana K, Foster C, O'Callaghan P, et al. Alterations in the metabolic and cardiorespiratory response to exercise in Huntington's Disease. Parkinsonism Relat Disord 2018; 54: 56–61.

Steventon JJ, Foster C, Furby H, Helme D, Wise RG, Murphy K. Hippocampal Blood Flow Is Increased After 20 min of Moderate-Intensity Exercise. 2019: 1–9.

Ströhle A, Schmidt DK, Schultz F, Fricke N, Staden T, Hellweg R, et al. Drug and Exercise Treatment of Alzheimer Disease and Mild Cognitive Impairment: A Systematic Review and Meta-analysis of Effects on Cognition in Randomized Controlled Trials. Am J Geriatr Psychiatry 2015; 23: 1234–1249.

Teixeira CVL, Ribeiro de Rezende TJ, Weiler M, Magalhães TNC, Carletti-Cassani AFMK, Silva TQAC, et al. Cognitive and structural cerebral changes in amnestic mild cognitive impairment due to Alzheimer's disease after multicomponent training. Alzheimer's Dement Transl Res Clin Interv 2018; 4: 473–480.

Thomas SN, Schroeder T, Secher NH, Mitchell JH. Cerebral blood flow during submaximal

For Review Only

and maximal dynamic exercise in humans. J Appl Physiol 1989; 67: 744–8.

Vis JC, Nicholson LF, Faull RL, Evans WH, Severs NJ, Green CR. Connexin expression in Huntington's diseased human brain. Cell Biol Int 1998; 22: 837–47.

Vonsattel JP, DiFiglia M. Huntington disease. J Neuropathol Exp Neurol 1998; 57: 369–384.

Wall JT, Xu J, Wang X. Human brain plasticity: an emerging view of the multiple substrates and mechanisms that cause cortical changes and related sensory dysfunctions after injuries of sensory inputs from the body. Brain Res Brain Res Rev 2002; 39: 181–215.

Wang J, Alsop DC, Song HK, Maldjian JA, Tang K, Salvucci AE, et al. Arterial transit time imaging with flow encoding arterial spin tagging (FEAST). Magn Reson Med 2003; 50: 599–607.

Warnert EA, Murphy K, Hall JE, Wise RG. Noninvasive assessment of arterial compliance of human cerebral arteries with short inversion time arterial spin labeling. J Cereb Blood Flow Metab 2015; 35: 461–468.

Weiss T, Miltner WHR, Liepert J, Meissner W, Taub E. Rapid functional plasticity in the primary somatomotor cortex and perceptual changes after nerve block. Eur J Neurosci 2004; 20: 3413–23.

Weng TB, Pierce GL, Darling WG, Falk D, Magnotta VA, Voss MW. The Acute Effects of Aerobic Exercise on the Functional Connectivity of Human Brain Networks. Brain Plast (Amsterdam, Netherlands) 2017; 2: 171–190.

West BT, Galecki AT. An Overview of Current Software Procedures for Fitting Linear Mixed Models. Am Stat 2012; 65: 274–282.

Winter B, Breitenstein C, Mooren FC, Voelker K, Fobker M, Lechtermann A, et al. High impact running improves learning. Neurobiol Learn Mem 2007; 87: 597–609.

For Review Only

Wolf RC, Grön G, Sambataro F, Vasic N, Wolf ND, Thomann PA, et al. Magnetic resonance perfusion imaging of resting-state cerebral blood flow in preclinical Huntington's disease. J Cereb Blood Flow Metab 2011; 31: 1908–18.

Wolf RC, Sambataro F, Vasic N, Schönfeldt-Lecuona C, Ecker D, Landwehrmeyer B. Aberrant connectivity of lateral prefrontal networks in presymptomatic Huntington's disease. Exp Neurol 2008; 213: 137–144.

Wolf RC, Vasic N, Schönfeldt-Lecuona C, Landwehrmeyer GB, Ecker D. Dorsolateral prefrontal cortex dysfunction in presymptomatic Huntington's disease: evidence from event-related fMRI. Brain 2007; 130: 2845–57.

Wong EC, Buxton RB, Frank LR. Implementation of quantitative perfusion imaging techniques for functional brain mapping using pulsed arterial spin labeling. NMR Biomed 1997; 10: 237–49.

Wong EC, Buxton RB, Frank LR. Quantitative imaging of perfusion using a single subtraction (QUIPSS and QUIPSS II). Magn Reson Med 1998; 39: 702–708.

Wu W-C, Jiang S-F, Yang S-C, Lien S-H. Pseudocontinuous arterial spin labeling perfusion magnetic resonance imaging--a normative study of reproducibility in the human brain. Neuroimage 2011; 56: 1244–50.

Zappe AC, Reichold J, Burger C, Weber B, Buck A, Pfeuffer J, et al. Quantification of cerebral blood flow in nonhuman primates using arterial spin labeling and a two-compartment model. Magn Reson Imaging 2007; 25: 775–83.

For Review Only

Legends for figures

FIGURE 1 STUDY DESIGN. MRI MEASURES WERE RECORDED UP TO 65-MINUTES AFTER EXERCISE CESSATION. ASL: ARTERIAL SPIN LABELLING MRI. T_1-W: T_1-WEIGHTED STRUCTURAL MRI SCAN ACQUIRED FOR IMAGE REGISTRATION PURPOSES. CYCLING WAS PERFORMED ON AN UPRIGHT ERGOMETER. * A SCAN TO MEASURE CEREBROVASCULAR REACTIVITY (CVR) USING A BREATH-HOLD DESIGN WAS ACQUIRED BUT NOT ANALYSED DUE TO POOR PERFORMANCE.

FIGURE 2 ABSOLUTE CHANGE IN CEREBRAL BLOOD FLOW (CBF) AND END-TIDAL CO2 (BOTTOM RIGHT PANEL) FROM BASELINE, MEASURED AT 15-, 40-, AND 60-MINUTES FOLLOWING EXERCISE CESSATION. DATA SHOWN ARE THE MARGINAL MEANS ADJUSTED FOR $P_{ET}CO_2$, SEX AND AGE. ERROR BARS REPRESENT THE STANDARD ERROR OF THE MEAN. MFG: MIDDLE FRONTAL GYRUS * $P < 0.05$ GENOTYPE EFFECT. † $P < 0.05$, †† $P < 0.01$ MAIN EFFECT OF EXERCISE.

FIGURE 3. LINEAR MODEL PREDICTORS OF PERFUSION CHANGE 40-MINUTES AFTER EXERCISE CESSATION. HRR: HEART RATE RESERVE DURING EXERCISE. TMS: TOTAL MOTOR SCORE. SCALED COEFFICIENT ESTIMATES, 95% CONFIDENCE INTERVALS AND COEFFICIENT OF UNCERTAINTY SHOWN. RESULTS OF STEPWISE REGRESSION FOR PREDICTORS: * $P < 0.05$, *** $P < 0.001$

For Review Only

TABLE 1 SOCIODEMOGRAPHIC, FITNESS, GENETIC AND CLINICAL DATA FOR HD AND CONTROL PARTICIPANTS. MEAN ± STANDARD ERROR MEAN (RANGE). ONE-WAY ANOVA OR CHI-SQUARED TESTS WERE USED TO COMPARE HD PATIENTS TO CONTROLS. IPAQ: INTERNATIONAL PHYSICAL ACTIVITY QUESTIONNAIRE. MAP: MEAN ARTERIAL PRESSURE. † REST MEASURES CALCULATED AFTER 15-MINUTES SUPINE REST.

	HD (n = 19)	Healthy controls (n=19)	*p-value*
Gender (male, N)	13	10	0.32
Age	45.9 ± 2.2 (32-65)	42.2 ± 2.1 (29-63)	0.23
BMI	26.2 ± 1.3 (18-40)	27.6 ± 0.9 (22-40)	0.41
IPAQ	23299.0 ± 20591.2 (0-352512)	2807.4 ± 682.8 (396-13488)	0.34
VO_2 peak (ml/kg/min)	34.6 ± 2.5 (18-50)	37.4 ± 2.3 (25-61)	0.42
MAP (mmHg)†	92.70 ± 2.07	88.06 ± 2.77	0.19
CAG repeat length	43.7 ± 0.6 (41-50)	N/A	
Disease burden [(CAG $length_n$-35.5) x age]	370.3 ± 24.4 (208-575)		
UHDRS Total Motor Score	25.7 ± 4.4 (0-68)		
UHDRS composite score (Schobel *et al.*, 2017)	12.13 ±1.19 (2.8-18.6)		
UHDRS Total Functioning Capacity	10.2 ± 0.8 (3-13)		

For Review Only

Table 2 Estimated marginal means (± S.E.M) after adjusting for $PETCO_2$, age and sex for cardiorespiratory and cerebrovascular measures at baseline and after 20-minutes of moderate intensity exercise. CON= healthy controls. Data in bold represent a significant effect of exercise in post-hoc analyses.

	Baseline		Post exercise scan session					
			Post 1		Post 2		Post 3	
	CON	HD	CON	HD	CON	HD	CON	HD
Cardiorespiratory measures								
HR, beats/min	62.0 ± 2.2	69.8 ± 2.6	**69.9 ± 2.7**	**77.8 ± 2.5**	**65.2 ± 2.2**	72.2 ± 2.1	64.5 ± 2.6	74.2 ± 2.7
MAP, mmHg	87.5 ± 2.2	92.7 ± 2.1	86.9 ± 2.5	90.2 ± 2.3	87.2 ± 3.0	89.9 ± 2.9	87.9 ± 2.8	92.5 ± 2.6
$PETCO_2$, mmHg	36.2 ± 1.2	34.7 ± 1.4	**34.4 ± 1.2**	**31.6 ± 1.3**	**34.0 ± 1.2**	**30.3 ± 1.7**	**34.6 ± 1.1**	**32.0 ± 1.5**
Perfusion (ml/100g/min) in ROIs								
Grey matter	52.8 ± 2.9	51.5 ± 3.2	53.3 ± 3.0	55.6 ± 3.2	50.2 ± 3.0	56.1 ± 3.4	52.0 ± 3.0	53.5 ± 3.4
Middle frontal gyrus	43.4 ± 4.2	38.8 ± 4.5	43.5 ± 4.4	41.5 ± 4.6	42.3 ± 4.4	**51.4 ± 5.1**	42.8 ± 4.6	39.1 ± 5.1
Postcentral gyrus	51.0 ± 4.7	48.5 ± 5.0	52.7 ± 4.8	54.9 ± 5.2	51.4 ± 4.8	60.9 ± 5.6	55.0 ± 4.9	54.1 ± 5.7
Precentral gyrus	54.4 ± 4.1	51.7 ± 4.4	52.8 ± 4.2	56.3 ± 4.5	50.2 ± 4.2	**61.6 ± 4.9**	51.5 ± 4.4	55.5 ± 4.9
Caudate	31.6 ± 3.1	24.3 ± 2.3	29.7 ± 2.3	26.1 ± 4.2	29.7 ± 2.9	26.1 ± 2.5	37.0 ± 6.8	25.7 ± 3.4
Thalamus	49.5 ± 4.8	41.1 ± 3.6	49.1 ± 3.4	42.8 ± 4.4	45.8 ± 4.4	46.3 ± 5.0	41.7 ± 3.1	40.7 ± 4.4
Hippocampus	51.0 ± 4.7	48.5 ± 5.0	52.7 ± 4.8	54.9 ± 5.2	51.4 ± 4.8	**60.9 ± 5.6**	55.0 ± 4.9	54.1 ± 5.7

For Review Only

TABLE 3 COGNITIVE PERFORMANCE AT BASELINE AND APPROXIMATELY 75-MINUTES AFTER EXERCISE CESSATION. MEANS ± S.E.M. DATA ARE MARGINAL MEANS ADJUSTED FOR AGE, AND FOR THE SYMBOL DIGIT SCORE, SCOLP AND TRAIL MAKING, ALSO ADJUSTED FOR MOTOR SPEED.

	Controls		HD		FDR-adjusted p values	
	Baseline	Post exercise	Baseline	Post exercise	Exercise	Gene Status
Symbol digit score	61.3 ± 2.9	57.2 ± 3.0	41.8 ± 3.1	40.4 ± 3.2	**0.015**	**0.0004**
SCOLP	84.5 ± 4.8	78.6 ± 4.9	52.1 ± 5.1	50.1 ± 5.2	**0.012**	**0.0004**
Digit Span (raw)	11.7 ± 0.5	11.8 ± 0.6	9.7 ± 0.6	9.1 ± 0.6	0.3	**0.004**
Trail Making (s)	44.7 ± 11.7	51.3 ± 12.2	96.9 ± 11.8	106.8 ± 13.1	0.14	**0.0053**
Verbal Fluency (1 letter)	17.8 ± 1.2	15.5 ± 1.2	9.7 ± 1.2	10.3 ± 1.2	0.26	**0.0004**
Stroop Interference	50.1 ± 3.1	44.8 ± 3.1	36.0 ± 3.13	33.2 ± 3.1	**0.012**	**0.005**
Motor Speed (taps/min)	452 ± 20.9	459 ± 21.3	337 ± 21.4	331 ± 21.3	0.96	**0.0004**

For Review Only

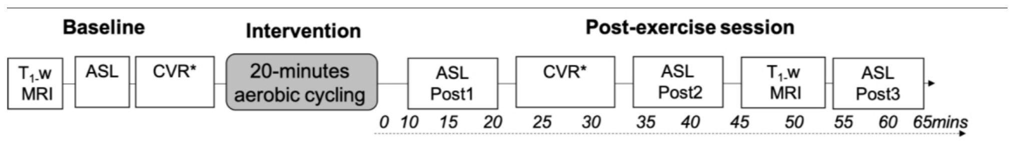

Caption : FIGURE 1 STUDY DESIGN. MRI MEASURES WERE RECORDED UP TO 65-MINUTES AFTER EXERCISE CESSATION. ASL: ARTERIAL SPIN LABELLING MRI. T1-W: T1-WEIGHTED STRUCTURAL MRI SCAN ACQUIRED FOR IMAGE REGISTRATION PURPOSES. CYCLING WAS PERFORMED ON AN UPRIGHT ERGOMETER. * A SCAN TO MEASURE CEREBROVASCULAR REACTIVITY (CVR) USING A BREATH-HOLD DESIGN WAS ACQUIRED BUT NOT ANALYSED DUE TO POOR PERFORMANCE.

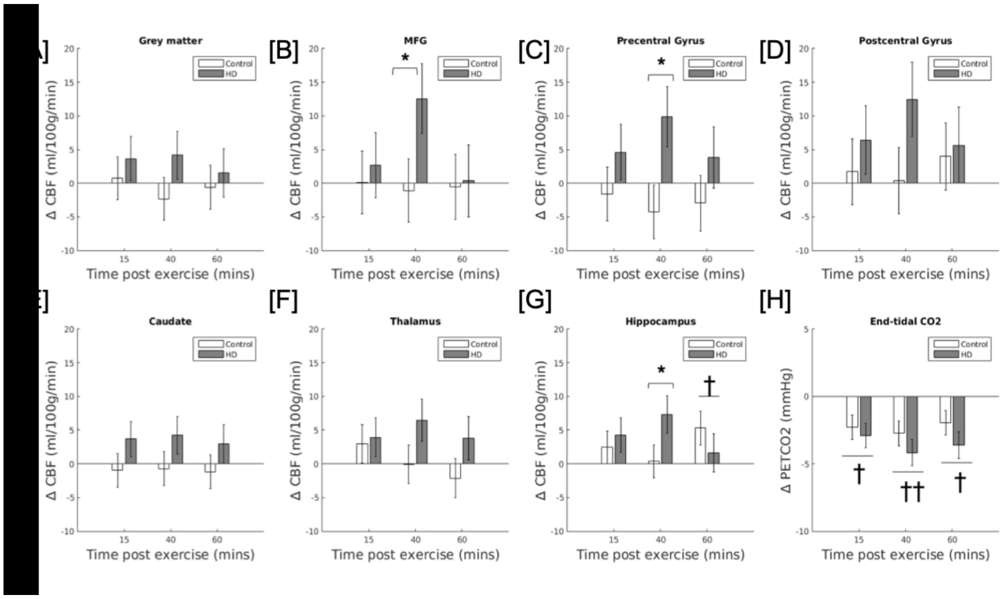

FIGURE 2 ABSOLUTE CHANGE IN CEREBRAL BLOOD FLOW (CBF) AND END-TIDAL CO2 (BOTTOM RIGHT PANEL) FROM BASELINE, MEASURED AT 15-, 40-, AND 60-MINUTES FOLLOWING EXERCISE CESSATION. DATA SHOWN ARE THE MARGINAL MEANS ADJUSTED FOR PETCO2, SEX AND AGE. ERROR BARS REPRESENT THE STANDARD ERROR OF THE MEAN. MFG: MIDDLE FRONTAL GYRUS * $P < 0.05$ GENOTYPE EFFECT. † $P < 0.05$, †† $P < 0.01$ MAIN EFFECT OF EXERCISE.

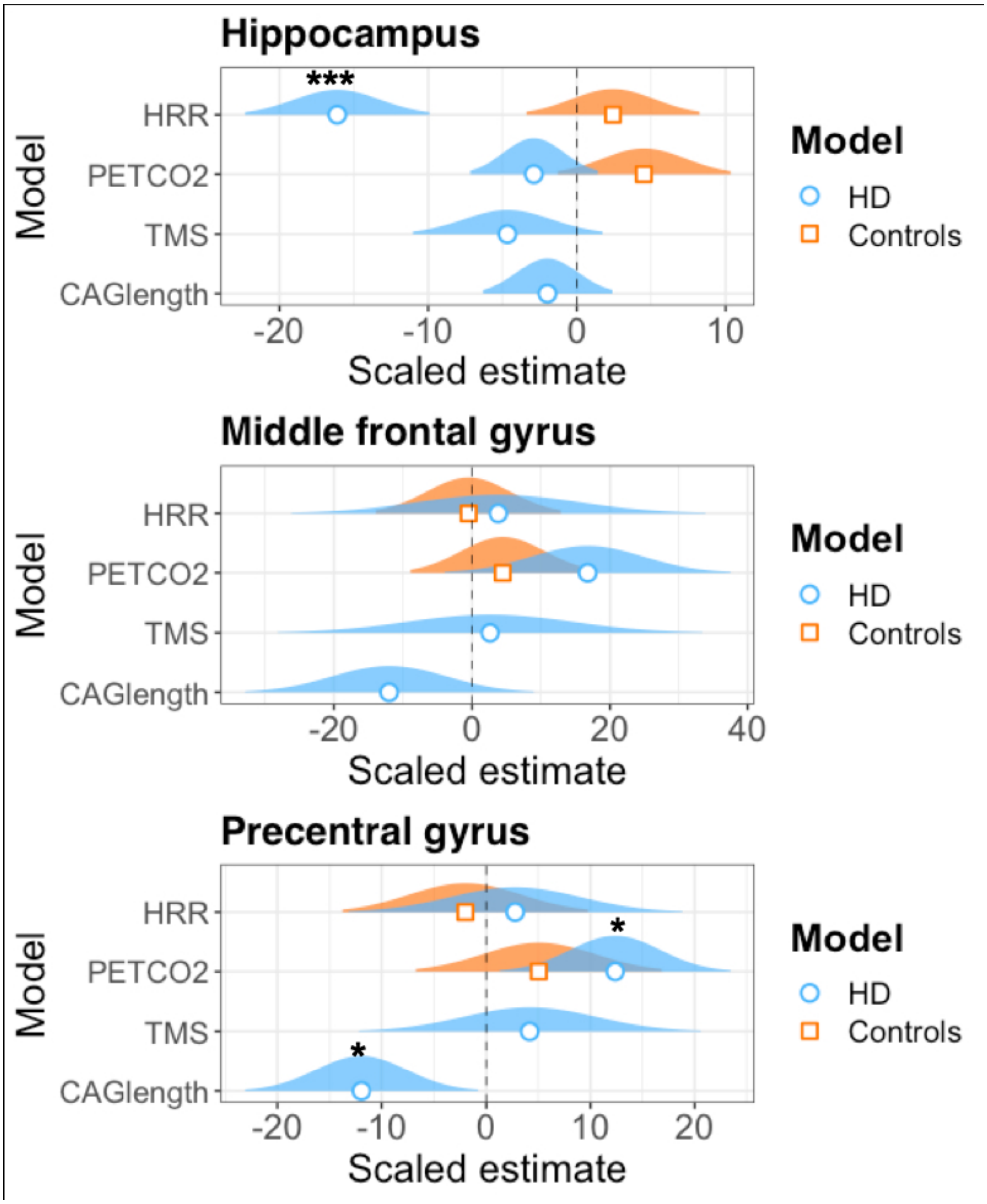

FIGURE 3. LINEAR MODEL PREDICTORS OF PERFUSION CHANGE 40-MINUTES AFTER EXERCISE CESSATION. HRR: HEART RATE RESERVE DURING EXERCISE. TMS: TOTAL MOTOR SCORE. SCALED COEFFICIENT ESTIMATES, 95% CONFIDENCE INTERVALS AND COEFFICIENT OF UNCERTAINTY SHOWN. RESULTS OF STEPWISE REGRESSION FOR PREDICTORS: * P <0.05, *** P < 0.001
