# Supplementary material for: Altered cerebrovascular response to acute exercise in patients with Huntington’s disease
Source: Brain Commun. 2020 Apr 16;2(1):fcaa044. doi: 10.1093/braincomms/fcaa044 (PMC7293798; doi:10.1093/braincomms/fcaa044)
Supplement: fcaa044_Supplementary_Data [file fcaa044_supplementary_data.zip › Supplementary Data.docx]

# Supplementary Data

### Cerebrovascular reactivity breath-hold challenge

During the CVR breath-hold challenge, participants were instructed to complete 10 breath-holds. A successful breath-hold performance was defined as: (a) a normalised PETCO_2_ peak from Fourier transform over the PETCO_2_ time course greater than or equal to 10, and (b) increased PETCO_2_ on exhalation following the breath-hold on more than half of the trials.

Analysis of the end-tidal CO2 traces show performance was poor in both HD participants (5.4 ± 0.8 successful breath holds) and control participants (4.8 ± 0.8 successful breath holds); 11 HD participants and 15 control participants did not meet the criteria for a successful breath-hold challenge, whilst two controls and three HD participants did not complete the challenge at both timepoints.

Supplementary Table 1 Unadjusted values for cardiorespiratory and cerebral measures, stratified for gene status and time relative to the exercise intervention. HR: heart rate. MAP: mean arterial pressure, GM: grey matter, MFG: middle frontal gyrus

|  | **Baseline** | | | |  | **Post 1** | | | |  | **Post 2** | | | |  | **Post 3** | | | |
| --- | --- | --- | --- | --- | --- | --- | --- | --- | --- | --- | --- | --- | --- | --- | --- | --- | --- | --- | --- |
|  | **Controls** | | **HD** | |  | **Controls** | | **HD** | |  | **Controls** | | **HD** | |  | **Controls** | | **HD** | |
|  | **Mean** | **SD** | **Mean** | **SD** |  | **Mean** | **SD** | **Mean** | **SD** |  | **Mean** | **SD** | **Mean** | **SD** |  | **Mean** | **SD** | **Mean** | **SD** |
| HR, beats/min | 62.0 | 9.8 | 69.8 | 11.3 |  | 69.9 | 10.2 | 77.8 | 11.4 |  | 65.2 | 9.2 | 72.2 | 9.4 |  | 64.5 | 10.2 | 74.2 | 10.9 |
| PETCO_2_, mmHg | 36.3 | 5.1 | 34.9 | 5.6 |  | 34.4 | 4.8 | 31.6 | 5.3 |  | 34.0 | 4.9 | 31.3 | 5.4 |  | 34.6 | 4.4 | 32.0 | 5.3 |
| MAP, mmHg | 88.1 | 12.1 | 92.7 | 9.0 |  | 86.9 | 12.3 | 90.2 | 7.8 |  | 87.2 | 14.2 | 89.9 | 11.2 |  | 87.9 | 12.8 | 92.5 | 9.7 |
| ***CBF (ml/100g/min)*** | | | | | | | | | | | | | | | | | | | |
| GM | 53.2 | 15.0 | 49.6 | 11.0 |  | 53.3 | 11.5 | 52.0 | 12.8 |  | 50.9 | 17.2 | 52.1 | 11.0 |  | 53.2 | 12.8 | 49.2 | 11.3 |
| Precentral Gyrus | 55.3 | 17.0 | 49.5 | 16.1 |  | 52.2 | 13.7 | 52.4 | 16.6 |  | 50.4 | 17.5 | 55.9 | 17.6 |  | 52.2 | 17.3 | 49.8 | 16.8 |
| Postcentral Gyrus | 52.3 | 17.8 | 45.4 | 18.6 |  | 52.0 | 14.2 | 49.7 | 16.8 |  | 51.4 | 21.1 | 53.2 | 18.7 |  | 56.1 | 25.6 | 46.7 | 17.1 |
| MFG | 44.9 | 15.6 | 36.4 | 18.0 |  | 42.4 | 11.9 | 37.8 | 16.1 |  | 42.3 | 21.0 | 43.3 | 21.1 |  | 42.9 | 16.8 | 34.7 | 17.0 |
| Hippocampus | 48.7 | 13.8 | 48.7 | 12.9 |  | 49.9 | 12.8 | 51.0 | 17.2 |  | 48.6 | 15.5 | 54.6 | 13.2 |  | 54.2 | 9.8 | 47.9 | 12.0 |
| Thalamus | 45.3 | 13.4 | 40.8 | 13.7 |  | 45.6 | 16.7 | 42.9 | 17.4 |  | 42.7 | 19.0 | 45.9 | 18.8 |  | 41.7 | 11.7 | 40.7 | 16.6 |
| Caudate | 30.5 | 12.6 | 24.3 | 9.0 |  | 29.2 | 9.4 | 26.0 | 16.2 |  | 29.6 | 11.9 | 26.0 | 9.6 |  | 29.4 | 13.1 | 25.0 | 12.3 |
| **Arterial arrival time (seconds)** | | | | | | | | | | | | | | | | | | | |
| GM | 0.75 | 0.03 | 0.74 | 0.03 |  | 0.74 | 0.04 | 0.74 | 0.04 |  | 0.74 | 0.04 | 0.72 | 0.04 |  | 0.74 | 0.06 | 0.74 | 0.04 |
| Precentral | 0.87 | 0.08 | 0.84 | 0.09 |  | 0.86 | 0.09 | 0.85 | 0.10 |  | 0.85 | 0.09 | 0.80 | 0.10 |  | 0.83 | 0.11 | 0.83 | 0.08 |
| Postcentral | 0.85 | 0.09 | 0.84 | 0.09 |  | 0.85 | 0.09 | 0.85 | 0.10 |  | 0.85 | 0.09 | 0.79 | 0.09 |  | 0.82 | 0.11 | 0.81 | 0.08 |
| MFG | 0.91 | 0.11 | 0.84 | 0.13 |  | 0.88 | 0.10 | 0.89 | 0.13 |  | 0.89 | 0.10 | 0.84 | 0.14 |  | 0.88 | 0.14 | 0.83 | 0.10 |
| Hippocampus | 0.66 | 0.04 | 0.67 | 0.04 |  | 0.66 | 0.04 | 0.66 | 0.04 |  | 0.65 | 0.05 | 0.65 | 0.05 |  | 0.66 | 0.04 | 0.67 | 0.04 |
| Thalamus | 0.79 | 0.07 | 0.77 | 0.07 |  | 0.77 | 0.07 | 0.78 | 0.07 |  | 0.76 | 0.08 | 0.75 | 0.09 |  | 0.76 | 0.10 | 0.79 | 0.07 |
| Caudate | 0.72 | 0.05 | 0.71 | 0.06 |  | 0.73 | 0.08 | 0.73 | 0.07 |  | 0.73 | 0.06 | 0.72 | 0.09 |  | 0.71 | 0.06 | 0.73 | 0.06 |


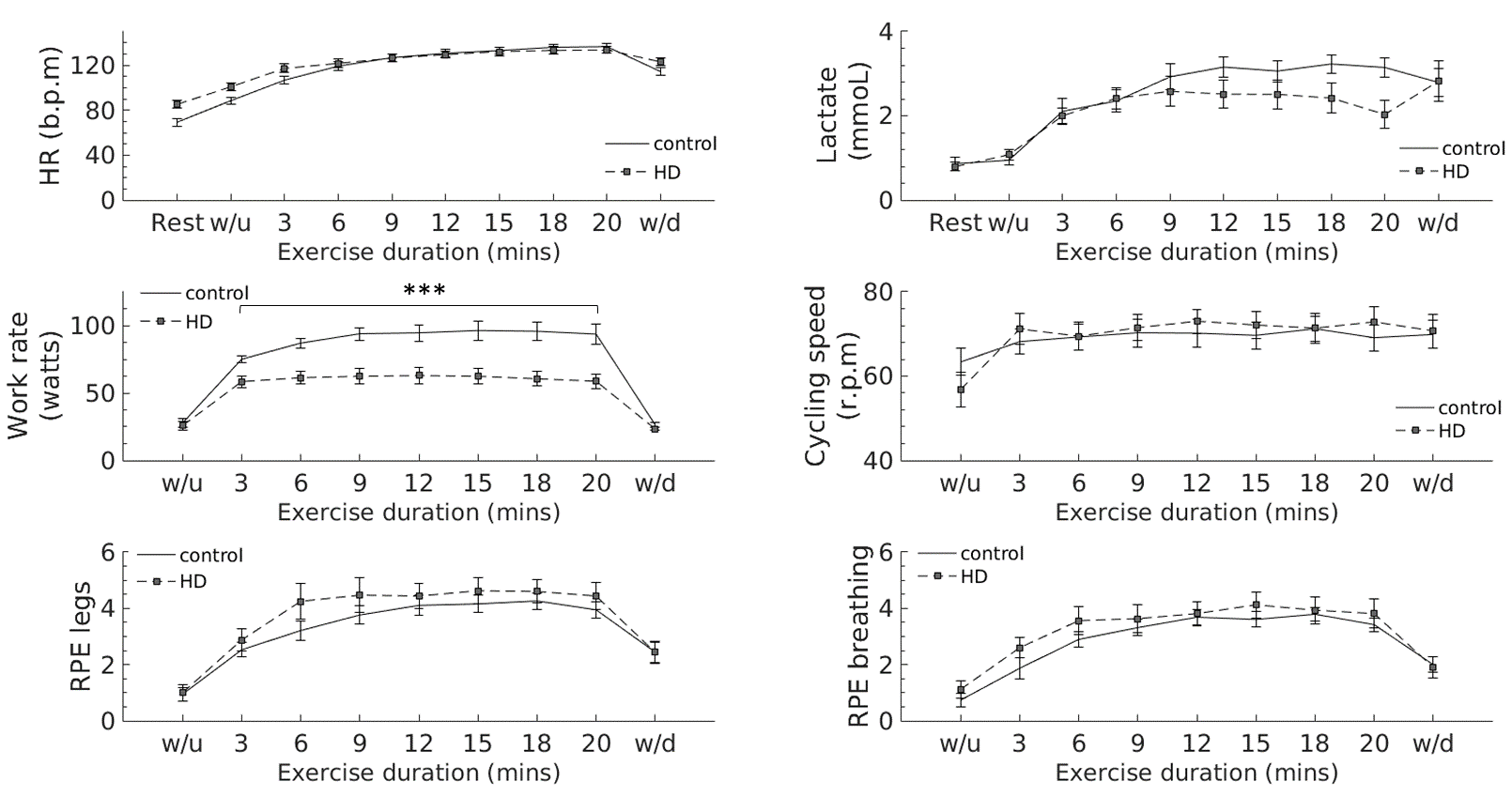


Supplementary Figure 1. Physiological, perceptual and performance response to the 20-minute exercise intervention. w/u : warm-up period, w/d: warm-down period. HR: heart rate; RPE: rating of perceived exertion [10-point scale]. Data are means ± S.E.M. *** p < 0.001.
