# Supplementary material for: Altered cerebrovascular response to acute exercise in patients with Huntington’s disease
Source: Brain Commun. 2020 Apr 16;2(1):fcaa044. doi: 10.1093/braincomms/fcaa044 (PMC7293798; doi:10.1093/braincomms/fcaa044)
Supplement: fcaa044_Supplementary_Data [file Original_submission_decision_letter.pdf › Original_submission_decision_letter.pdf]

BRAINCOM-2019-192
6th February 2020

Dear Dr. Steventon,

Thank you for your recent submission BRAINCOM-2019-192 entitled "Altered cerebrovascular response to acute exercise in patients with Huntington's Disease" to Brain Communications.

Your manuscript has been reviewed, and we apologize for the delay in securing the necessary number of reviewers. While the reviewers and editorial team appreciate many aspects of the paper, they raise some concerns that you will need to address in order for the paper to be considered for publication. In particular, the relevance of the findings for HD needs to be addressed in more detail.

Therefore, I invite you to respond to the reviewer(s)' comments and revise your manuscript. The reviewer(s)' comments are included at the bottom of this message.

In addition to the reviewers' comments, please note our editorial comments added below to comply with our requirements for presentation of scientific data at Brain Communications.

Please format your revised manuscript according to our Instructions to Authors https://academic.oup.com/braincomms/pages/General_Instructions – a summary of key points is attached to help with this.

To revise your manuscript, click the link below or log into https://mc.manuscriptcentral.com/braincom and enter your Author Centre, where you will find your manuscript title listed under "Manuscripts with Decisions." Under "Actions," click on "Create a Revision." Your manuscript number has been appended to denote a revision.

*** PLEASE NOTE: This is a two-step process. After clicking on the link, you will be directed to a webpage to confirm. ***

https://mc.manuscriptcentral.com/braincom?URL_MASK=a37668efb3d74f60a1ef0f6c6a886ef6

Please upload a revised manuscript with changes tracked with track changes or in coloured text.

When submitting your revised manuscript, you will be asked to respond to the reviewer(s) comments. Please be as specific as possible in your response to all of the comments.

The editorial team at Brain Communications aims to increase transparency in scientific publishing and to demonstrate the value that peer review brings to the scientific process. Please therefore consider whether you are willing to have reviewer(s)' comments on your paper and your response published as supplementary data with the paper if it is accepted. This is entirely optional and will only be done if both the authors and reviewers agree. While submitting your revision there will be an opportunity to review or update the answer you gave previously, if necessary.

IMPORTANT: Your original files are available to you when you upload your revised manuscript. Please delete any redundant files before completing the submission.

Because we are trying to facilitate timely publication of manuscripts submitted to Brain Communications, we would appreciate your revised manuscript as soon as possible. If it is not possible for you to resubmit within 3 months, the option to submit a revision will expire, and we would then consider your revised paper as a new submission. However, if you need a little more time, please get in touch and we can extend this deadline for you.

Once again, thank you for submitting your manuscript to Brain Communications and I look forward to receiving your revision.

Yours sincerely,
Dr. Josef Priller
Editor, Brain Communications

Reviewer(s)' Comments to Author:

Reviewer: 1

Comments to the Author
This in an interesting study examining the effects of acute exercise on specific brain imaging measures in subjects gene-positive for the Huntington's disease (HD) gene mutation, and matched controls. Whilst the study is novel and the findings generally well described, I have a number of suggestions below for improvements to the manuscript.

1. The study would be much more informative, with respect to HD, if the authors were able to have an adequately powered gene-positive presymptomatic group (currently n=7) approximately matched to the symptomatic HD group (n=12) and control (n=19) group sizes. Any data on a well powered gene-positive pre-manifest group would provide insight into whether these abnormal brain changes occur prior to symptom onset, and thus might be more likely to be causally involved in early pathogenesis of HD.

2. The Statistical analyses states that 'An initial quality assessment removed any statistical outliers defined as more than 3 standard deviations from the mean'. I think it is always better not to remove outliers, and their removal needs strong justification. Often such outliers can be highly informative, if the investigators can establish why they are outliers. What does the data look like if no outliers are removed.

3. In Figure 3, in precentral gyrus, CAG length predicts the change. Can the authors explain why this is not the case in other brain regions?

4. Could the change in cognitive performance be due to fatigue? Or the exercise program might otherwise negatively impact on the participants' cognitive function?

5. The acute exercise might be a stressor, and this could inform the mechanisms behind the data reported in this manuscript. Could the authors provide data on cortisol, and/or other stress responses? The authors could at the very least discuss in detail the evidence that the HPA-axis is altered both in clinical HD and preclinical models, and that in at least one preclinical model stress (and increased stress hormone) can exacerbate cognitive deficits and other specific symptoms.

6. There is also evidence of cardiovascular changes (including the heart itself) in preclinical models and clinical HD, and this worth discussing in more detail.

7. There are a couple spots where the citations were misformatted as "Error! Reference source not found..". The whole manuscript should be proofread again.

Reviewer: 2

Comments to the Author
This study investigates the effects of acute exercise on the cerebral perfusion. The authors have identified a transient alteration in cerebral blood flow in HD compared to controls after forty minutes of exercise, suggestive of a disturbed cerebrovasculature.
This is an interesting, well-conducted study that shows potential for the role of vascular changes in HD. However, given the inevitable heterogeneity within such a small HD sample, any findings need to be tempered with considerable caution. Further investigation into vascular changes in HD is really necessary before making any of the conclusions presented here.
Methods
The age range is large, suggesting that there is considerable heterogeneity in terms of disease stage. This is reflected in the fact that in a small sample of 19 HD participants, there are preHD, Stage 1 and Stage 2 patients. This will likely impact the measured perfusion levels and changes in CBF with such variability making it more challenging to understand the overall findings. I appreciate that these are rare disease patients, but they are also quite phenotypically-heterogeneous even within the same disease stage.
Was TFC used for premanifest? This is not a very useful measure for this group.
IPAQ (as per the table) –huge variability in HD-related to movement problems? What impact could this have on the results?
Why not look at a priori regions not generally associated with HD eg hippocampus
What was the rationale behind the task selection?

What outliers were removed?
Results
Slightly confused re differences and no differences in heart rate between the groups. Please clarify.
Discussion
Results found in a priori chosen regions are not necessarily due to HD pathology – might have found something similar elsewhere in the brain if other ROIs had been investigated

Editorial Office comments:
-If your paper is accepted, we will require an abbreviated summary of no more than 50 words and a graphical abstract. Please include these in your revision.
