# Supplementary material for: Altered cerebrovascular response to acute exercise in patients with Huntington’s disease
Source: Brain Commun. 2020 Apr 16;2(1):fcaa044. doi: 10.1093/braincomms/fcaa044 (PMC7293798; doi:10.1093/braincomms/fcaa044)
Supplement: fcaa044_Supplementary_Data [file Original_submission_Response_to_reviewers.pdf › Original_submission_Response_to_reviewers.pdf]

Response to reviewers Brain Communications BRAINCOM-2019-192

Reviewer: 1

1. The study would be much more informative, with respect to HD, if the authors were able to have an adequately powered gene-positive presymptomatic group (currently n=7) approximately matched to the symptomatic HD group (n=12) and control (n=19) group sizes. Any data on a well powered gene-positive pre-manifest group would provide insight into whether these abnormal brain changes occur prior to symptom onset, and thus might be more likely to be causally involved in early pathogenesis of HD.

To ensure the study was adequately powered, we conducted an a priori power calculation which showed that we needed a total of 36 participants to achieve 80% power to detect a medium size effect ($F^2 = 0.3$), based on our previous work showing a medium effect size in HD in terms of the cardiometabolic response to exercise (Steventon et al, 2018). Thus, our priority was to recruit 2 groups of 20 participants (HD vs control, accounting for attrition / image quality issues) to ensure our statistical analysis was sufficiently powered to be able to draw inferences from the data.

We agree with the reviewer that the ability to recruit sufficient participant numbers in order to stratify across different stages of Huntington's disease with sufficient statistical power is desirable and represents an important future direction for the work, allowing us to answer novel questions about the relationship between disease stage in HD and brain function. However, the state of the current literature is that we do not know how HD per se affects blood flow in the brain, and the current study is the first of its kind to show differences in blood flow following exercise in HD participants. Given that HD is a rare disease and the unique study design imposed further restrictions on participation (contraindications to exercise, contraindications to MRI), recruitment of two adequately powered groups (pre-manifest and manifest) was not feasible in this study, especially with funding limitations due to MRI being an expensive research modality. The next incremental step would be to look at what stage in the disease these differences occur. This has been added to the discussion as a future direction and limitation.

"A limitation of our study is the heterogeneity of the HD group, which includes both presymptomatic and manifest gene carriers. Therefore, we were unable to identify at what stage in the disease an altered response to exercise occurs. Future work in a pre-symptomatic group is required to provide insight into whether the observed exercise-induced brain changes are causally involved in the early pathogenesis of HD."

Moreover, there is an argument to be made about the utility of stratifying groups according to manifest status in HD to understand the effect of disease on brain structure and function. Stratification based on premanifest versus symptomatic status does not reflect the functional heterogeneity in the disease, nor the non-binary nature of disease progression. The clinical diagnosis of HD is based on a diagnostic confidence score based on traditional motor criteria. This is based on the presentation in clinic on a single day and on only one symptom domain. HD is a functionally heterogenous disease, thus many people living with 'premanifest' HD are displaying clinically significant symptoms in other domains, such as cognitive and psychiatric symptoms. This is reflected in our current study where we had one participant with a TFC score indicative of disease stage 2, but a diagnostic confidence score < 4, indicating premanifest disease stage. For clarity, we have now used the diagnostic confidence for reporting premanifest or manifest, and TFC for disease stage in manifest participants only.

2. The Statistical analyses states that 'An initial quality assessment removed any statistical outliers defined as more than 3 standard deviations from the mean'. I think it is always better not to remove outliers, and their removal needs strong justification. Often such outliers can be highly informative, if the investigators can establish why they are outliers. What does the data look like if no outliers are removed.

We agree with the reviewer that outliers should not be removed without a justification / thorough investigation to determine if the outlier is plausible, as an outlier may provide a genuine observation and is not necessarily spurious simply by being an 'extreme' data point. We chose 3 standard deviations consistent with Tukey's criterion for extreme outliers. In the current analysis, only 1 outlier was identified for the MFG, precentral gyrus, hippocampus and caudate (out of 122 observations for MFG/precentral, and 242 observations for the caudate and hippocampus) and 2 outliers were found in the thalamus ROI analysis (both hemispheres). These identified outliers were from the same single control participant and were extreme across the ROIs for a single timepoint. This suggests that an error occurred in the preprocessing and/or quantification of CBF for this dataset. As well as examining the observed value in comparison to the group mean (inter-subject comparison), we also compared the value with the 3 previous CBF quantifications for that participant, as it is plausible that this participant has above average resting CBF. However, we found that CBF at this final timepoint was approximately double the observed blood flow at baseline, post-15, and post-40 minutes (ml/100g/min) which is biologically implausible in this situation. For a completely transparent analysis and reporting strategy the results with and without outlier inclusion can be found in the supplementary information. We have amended the sentence in the statistical analysis section of the methods to more accurately reflect the approach we took to outlier removal:
"An initial quality assessment examined any statistical outliers, defined as more than 3 standard deviations from the group mean, and removed them if found to be spurious (e.g. biologically implausible)."

3. In Figure 3, in precentral gyrus, CAG length predicts the change. Can the authors explain why this is not the case in other brain regions?

As the direction of the observed effects were in the opposite direction to what we hypothesised, we do not have a clear explanation for the relationship found between CAG length and CBF change in the precentral gyrus and are cautious to avoid over-interpreting the results. In paragraph 2 of the discussion, we have suggested this selective regional effect could reflect a compensatory mechanism to support neuronal survival, as it may be that certain regions are less vulnerable to hindered cerebral haemodynamics, however this is purely speculative and further research is required. In response to the reviewer's comment, we have made this explanation (and the association with CAG length) more explicit.

Based on the reviewer's comment, we have also expanded on this in the discussion and included previous work showing previous evidence of compensatory changes in the precentral gyrus, albeit in connectivity. The precentral gyrus is a key component of the motor network and both structural and functional changes in pre and early HD have been shown to be associated with motor dysfunction (e.g. Thu et al, 2020, Scahill et al, 2013, Dumas et al, 2012,Zimbelman et al., 2007) thus we know this area is implicated in HD pathology. Therefore, a dampened response to exercise in the precentral gyrus in participants with a larger CAG repeat length fits with this.
We did not look in all of the brain regions included in the analysis, only the brain regions where a significant effect was observed in our primary analysis, in order to reduce the number of planned comparisons. As can be seen in Figure 3, a similar trend was seen in the middle frontal gyrus however this was not significant.
.

4. Could the change in cognitive performance be due to fatigue? Or the exercise program might otherwise negatively impact on the participants' cognitive function?

Yes, we feel that the most likely explanation for the change in cognitive performance is fatigue. The explanation of the cognitive results is in paragraph 7 of the discussion, with fatigue presented as the most likely cause.

5. The acute exercise might be a stressor, and this could inform the mechanisms behind the data reported in this manuscript. Could the authors provide data on cortisol, and/or other stress responses? The authors could at the very least discuss in detail the evidence that the HPA-axis is altered both in clinical HD and preclinical models, and that in at least one preclinical model stress (and increased stress hormone) can exacerbate cognitive deficits and other specific symptoms.

Unfortunately, we do not have data on cortisol or other stress responses, other than the physiological responses included. The modulating effect of cortisol on cerebral blood flow is not well-established with mixed results – for example, Shilling et al (2004, doi: 10.1002/hbm.22304) directly tested this in human brain structures using ASL-MRI and found no effect of cortisol on regional blood flow, whereas in participants with schizophrenia, the administration of haloperidol, which reduces both IL-6 and cortisol, was associated with an increase in hippocampal rCBF, however this is in the opposite direction of our results. We have included a paragraph in the discussion including the evidence of an altered HPA-axis in HD and thank the reviewer for this helpful input.

"Exercise is a potent physiological stimulus upon the hypothalamo-pituitary adrenal (HPA) axis and there is evidence that the HPA axis is impaired in HD (Kassubek et al., 2004; Petersén et al., 2005; Björkqvist et al., 2006; Soneson et al., 2010; van Wamelen et al., 2014), with mixed evidence of subtle alterations in neuroendocrine signalling, including cortisol(Aziz et al., 2009; Kalliolia et al., 2015). There is equivocal evidence surrounding the modulating effect of cortisol on CBF(Hodkinson et al., 2014; Schilling et al., 2014; Handley et al., 2016) sufficient to merit future exercise studies in HD collecting neuroendocrine measures along with cardiorespiratory data. **"**

6. There is also evidence of cardiovascular changes (including the heart itself) in preclinical models and clinical HD, and this worth discussing in more detail.

We have previously shown an altered cardiorespiratory response to submaximal exercise in HD patients (Steventon *et al.*, 2018) and this is referred to in the introduction and is the basis for our hypothesis. Based on the reviewers comment, we have expanded on this and discussed the background work in preclinical models.
"It is plausible that cardiovascular changes observed in preclinical models and clinical HD(Wood et al., 2012; Buonincontri et al., 2014; Bellosta Diago et al., 2018; Steventon et al., 2018) may mediate the altered cerebral response observed. HD participants were found to have a higher heart rate across the study compared to controls, although no interaction effect was found with exercise. Future studies would combine comprehensive cardiovascular and cerebrovascular measures to investigate vascular impairments in HD in response to exercise."

7. There are a couple spots where the citations were misformatted as "Error! Reference source not found..". The whole manuscript should be proofread again.
These errors have been corrected.

Reviewer: 2

---

1. The age range is large, suggesting that there is considerable heterogeneity in terms of disease stage. This is reflected in the fact that in a small sample of 19 HD participants, there are preHD, Stage 1 and Stage 2 patients. This will likely impact the measured perfusion levels and changes in CBF with such variability making it more challenging to understand the overall findings. I appreciate that these are rare disease patients, but they are also quite phenotypically-

heterogeneous even within the same disease stage. Was TFC used for premanifest? This is not a very useful measure for this group.

As acknowledged by the reviewer, HD is a rare disease, and our study imposed additional restrictions on recruitment (contraindications to exercise, contraindications to MRI), and this is the primary reason for the heterogenous group. Recruitment is always a challenge is a rare disease cohort, especially when apathy is a clinical feature of the disease.
It is the case that this heterogeneity may impact of the ability to detect a group level effect in the ASL MRI data, however our a priori power calculation (80% power to detect a medium sized effect $F^2 = 0.3$) was based on data from a heterogenous HD group (Steventon et al, 2018) to ensure our statistical analysis was sufficiently powered to be able to draw inferences from the data.
The reviewer refers to the age range, however we took care to include age (along with sex and end-tidal CO2) in the statistical model and we do observe a difference in the perfusion response to exercise in the HD group, despite the heterogeneity.

We feel the functional heterogeneity in our cohort is representative of the disease; HD is a heterogenous disease, thus many people living with 'premanifest' HD are displaying clinically significant symptoms in other domains, such as cognitive and psychiatric symptoms. This is reflected in our current study where we had one participant with a TFC score indicative of disease stage 2, but a diagnostic confidence score < 4, indicating premanifest disease stage. For clarity, we have now used the diagnostic confidence for reporting premanifest or manifest, and TFC for disease stage in manifest participants only.

The heterogeneity in the HD group has been added to the discussion as a limitation.
"A limitation of our study is the heterogeneity of the HD group, which includes both presymptomatic and manifest gene carriers. Therefore, we were unable to identify at what stage in the disease an altered response to exercise occurs. Future work in a pre-symptomatic group is required to provide insight into whether the observed exercise-induced brain changes are causally involved in early pathogenesis of HD."

2. IPAQ (as per the table) –huge variability in HD-related to movement problems? What impact could this have on the results?

The IPAQ results, a self-reported measure of physical activity, was not correlated with total motor score on the UHDRS ($r = -0.16$, $p = 0.55$).
The variability the reviewer refers to in table 1 is due to an extreme value for a single highly active HD participant which was a statistical outlier (3.9 standard deviations from the mean, IPAQ total METS = 352512) - this participant had a very active job but did not have an extreme VO2 peak test, thus we decided to include the participant in the analysis. This extreme value was included in the range data in the table, however to avoid confusion, this data point has been removed from the group average in table 1 to avoid skewing the results and making a group comparison difficult for the reader, and instead is included as a footnote. As can be seen in the figure below, the variability now is comparable between HD and control participants.
Previous work has shown an inherent measurement error with the IPAQ with the majority of participants tending to under-report their levels of moderate-to-vigorous activity (https://doi.org/10.1186/s12874-018-0642-3) thus we have used the metric only to describe the groups in terms of background demographics, and also used an objective measure of cardiovascular fitness. With both measures, there was no statistical difference between groups which gives us confidence that the groups are well matched in this regard and therefore does not impact on any group differences observed.

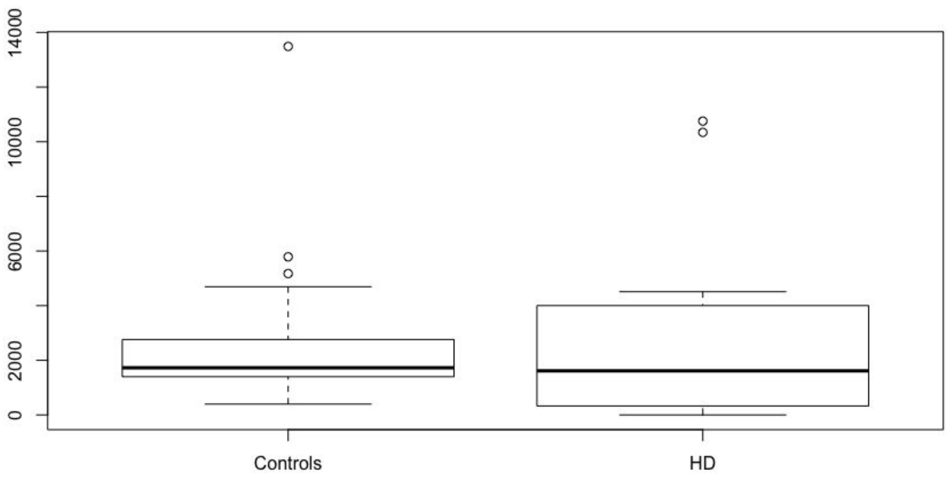

Figure 1 IPAQ scores for control and HD participants (after removing extreme value in HD group).

3. Why not look at a priori regions not generally associated with HD eg hippocampus

I'm not sure if the reviewer instead means 'why look at the hippocampus, as area not generally associated with HD' here. The hippocampus was an a priori region, based on previous work using the same research design which found a selective effect in the hippocampus in healthy participants. This is justified with appropriate references included in the methods section – the hippocampus has been shown to be selectively affected by exercise. It is often difficult to select a 'control' region (not associated with HD pathology) in HD studies as many brain areas are affected in HD with increased disease progression. The hippocampus has previously been shown to be impaired in HD (e.g. Begeti et al, 2016, DOI: 10.1136/jnnp-2015-312413; Spargo et al, 1993 found neuronal loss of 35% in the hippocampus, DOI: 10.1136/jnnp.56.5.487).

4. What was the rationale behind the task selection?

It is unclear what task the reviewer is referring to here.

The exercise intervention was chosen as it has been previously shown to induce changes in cerebral blood flow in healthy adults (Steventon et al, 2019, https://doi.org/10.1093/cercor/bhz104

5. What outliers were removed?

For the investigation of outliers, we chose 3 standard deviations consistent with Tukey's criterion for extreme outliers. Based on this, only 1 outlier was identified for the MFG, precentral gyrus, hippocampus and caudate (out of 122 observations for MFG/precentral, and 242 observations for the caudate and hippocampus) and 2 outliers were found in the thalamus ROI analysis (both hemispheres), defined as greater than 3 standard deviations from the mean. These identified outliers were from the same single control participant and were extreme across the ROIs for a single timepoint. This suggests that an error occurred in the pre-processing and/or quantification of CBF for this dataset. As well as examining the observed value in comparison to the group mean (inter-subject comparison), we also compared the value with the 3 previous CBF quantifications for that participant, as it is plausible that this participant has above average resting CBF. However, we found that CBF at this final timepoint was approximately double the observed blood flow at baseline, post-15, and post-40 minutes (ml/100g/min) which is biologically implausible in this situation. For a completely transparent analysis and reporting strategy the results with and without outlier inclusion can be found in the supplementary information. We have amended the sentence in the statistical analysis section of the methods to more accurately reflect the approach we took to outlier removal:

"An initial quality assessment examined any statistical outliers, defined as more than 3 standard deviations from the group mean, and removed them after investigation if found to be spurious (e.g. biologically implausible)."

6. Results. Slightly confused re differences and no differences in heart rate between the groups. Please clarify.

During the intervention period, we report a number of different results related to heart rate, including absolute change from rest, absolute heart rate (beats/min), and heart rate reserve. There was no difference between groups on any of these metrics. This has been clarified with a summary statement at the start of this section of the results.

Heart rate was also compared between the baseline and post-exercise scan sessions. HD participants had a higher heart rate overall compared to control participants, yet there was no interaction between exercise and genotype. This finding has been included in the discussion in the section focused on cardiovascular differences in HD.

7. Results found in a priori chosen regions are not necessarily due to HD pathology – might have found something similar elsewhere in the brain if other ROIs had been investigated

Our study design involved the recruitment of a gene-negative control group in order to be able to infer whether any observed changes were specific to HD. In a previous paper using the same experimental design with healthy controls we found a specific localised effect in the hippocampus (Steventon et al, 2019, https://doi.org/10.1093/cercor/bhz104) which was also found in this study. This suggests that this particular result is not due to HD pathology (and was found in both groups in this analysis).

The assessment of statistical power is imperative when designing studies with rare patient cohorts, due to the heterogeneity of patient groups and difficulties in recruiting. Whilst we agree that exercise-HD interactive effects may be present in other regions, given the small sample size (noted by the reviewer) it was necessary to select a number of a priori regions of interest to ensure we were sufficiently powered to detect an effect. We were insufficiently powered for a voxel-based approach which requires a large amount of multiple comparison corrections. The regions selected were based on the previous literature and are fully justified in the methods section.
